# A Jasmonate ZIM-Domain Protein NaJAZd Regulates Floral Jasmonic Acid Levels and Counteracts Flower Abscission in *Nicotiana attenuata* Plants

**DOI:** 10.1371/journal.pone.0057868

**Published:** 2013-02-28

**Authors:** Youngjoo Oh, Ian T. Baldwin, Ivan Galis

**Affiliations:** Department of Molecular Ecology, Max Planck Institute for Chemical Ecology, Jena, Germany; University of Delhi South Campus, India

## Abstract

Jasmonic acid is an important regulator of plant growth, development and defense. The jasmonate-ZIM domain (JAZ) proteins are key regulators in jasmonate signaling ubiquitously present in flowering plants but their functional annotation remains largely incomplete. Recently, we identified 12 putative JAZ proteins in native tobacco, *Nicotiana attenuata*, and initiated systematic functional characterization of these proteins by reverse genetic approaches. In this report, *Nicotiana attenuata* plants silenced in the expression of *NaJAZd* (irJAZd) by RNA interference were used to characterize NaJAZd function. Although *NaJAZd* transcripts were strongly and transiently up-regulated in the rosette leaves by simulated herbivory treatment, we did not observe strong defense-related phenotypes, such as altered herbivore performance or the constitutive accumulation of defense-related secondary metabolites in irJAZd plants compared to wild type plants, both in the glasshouse and the native habitat of *Nicotiana attenuata* in the Great Basin Desert, Utah, USA. Interestingly, irJAZd plants produced fewer seed capsules than did wild type plants as a result of increased flower abscission in later stages of flower development. The early- and mid-developmental stages of irJAZd flowers had reduced levels of jasmonic acid and jasmonoyl-L-isoleucine, while fully open flowers had normal levels, but these were impaired in *NaMYB305* transcript accumulations. Previously, *NaMYB305*-silenced plants were shown to have strong flower abscission phenotypes and contained lower *NECTARIN 1* transcript levels, phenotypes which are copied in irJAZd plants. We propose that the NaJAZd protein is required to counteract flower abscission, possibly by regulating jasmonic acid and jasmonoyl-L-isoleucine levels and/or expression of *NaMYB305* gene in *Nicotiana attenuata* flowers. This novel insight into the function of JAZ proteins in flower and seed development highlights the diversity of functions played by jasmonates and JAZ proteins.

## Introduction

Plants are frequently exposed to various abiotic and biotic stresses such as high light, water deficit, salinity stress, variable temperature, lack of nutrients, and attack from pathogens and herbivores. Survival of plants in nature thus strongly depends on a balance between growth and defense related processes, which is regulated by a complex phytohormone network [1]–[6]. In this network, jasmonic acid (JA) controls both growth and defense responses in plants (reviewed in [7]). JA is synthesized from membrane-derived fatty acids (18∶3) via the octadecanoid pathway [8] and is known to activate transcription factors (TFs) that trigger a large-scale transcriptional reprogramming of growth and development, such as root growth and adventitious root formation, trichome initiation, fruit ripening, anthocyanin accumulation, senescence, pollen and flower development, tuber formation and tendril coiling, and defense against wounding, herbivore attack and pathogen infection [9]–[13].

Recently, the mode of action and role of several core components in JA signaling, COI1 (CORNATINE INSENSITIVE1), JAZ (Jasmonate ZIM-domain), and (+)-*7*-*iso*-JA-L-Ile (JA-Ile) were identified [14]–[18]. In the presence of the active hormone, JA-Ile, JAZ proteins are degraded by the action of SCF^COI1^-E3 ubiquitin ligase complex associated with 26S proteasome that releases the positive regulators of JA signaling, MYC2/3/4 transcription factors and triggers the expression of JA-dependent genes in *Arabidopsis* (reviewed in [19]). In addition, the function of several co-regulators of the core complex of JA signaling, such as NINJA (Novel Interactor of JAZ) and TPL (TOPLESS) proteins, InsP_5_ (inositol pentakisphosphate), EIN3/EIL1 (ethylene-stabilized transcription factors), R2R3-MYB transcription factors MYB21 and MYB24, WD-repeat/bHLH (GL3, EGL3, TT8)/MYB75 complexes and DELLA proteins were elucidated [20]–[25].

JAZ proteins that are generally classified as negative regulators of JA signaling contain two functionally conserved domains, ZIM with TIF[F/Y]XG motif (or its variant) and Jas with S-L-X(2)-F-X(2)-K-R-X(2)-R motifs, both of which are essential for JA signal transduction [26]–[29]. ZIM domains mediate the homo- and heteromeric interactions among the JAZ proteins as well as their interaction with the co-repressor NINJA-TPL complex; the Jas domain is required for binding several core- (COI1, MYC2/3/4) and co- (EIN3/EIL1, MYB21/24, TT8/GL3/EGL3 and DELLA) regulatory proteins that transduce downstream signaling (reviewed in [19]). It was proposed that different combinations and interactions between JAZ proteins and co-regulators can control specific subsets of JA-mediated responses in plants [30], [31] however, specific examples of such interactions remain rare. Functional studies with genetically modified plants have provided evidence of the direct involvement of JA and JAZ proteins in developmental processes such as secondary growth (interfascicular cambium initiation) [32], phytochrome A-mediated shade responses [33], anthocyanin accumulation and trichome initiation [24], stamen development [23], flower induction [34], and defense responses against biotic [31], [35]–[37] and abiotic [38]–[41] stresses. However, additional experiments are required to better understand the complex networking among JA, JAZ, and downstream responses in plants.

Previously, we cloned 12 novel *JAZ* genes from the native tobacco plant *Nicotiana attenuata* (*N. attenuata*) and reported unique roles for NaJAZh in defense and development [31]. Here, we examine the function of NaJAZd, both in development and defense against herbivores. The *NaJAZd*-silenced plants had normal levels of defense-related phytohormones and only slightly altered defense metabolic profiles in the leaves. In development, irJAZd plants had significantly impaired seed production which is one of the most important fitness parameters in *N. attenuat*a plants. We show that NaJAZd is involved in the regulation of flower abscission which in turn is associated with reduced jasmonate levels and impaired expression of genes (*NaMYB305*, *NaNEC1*) known to be important for flower development.

## Results

### NaJAZd Transcript Accumulation is Strongly Induced by Wounding and Herbivory

**Figure 1:**
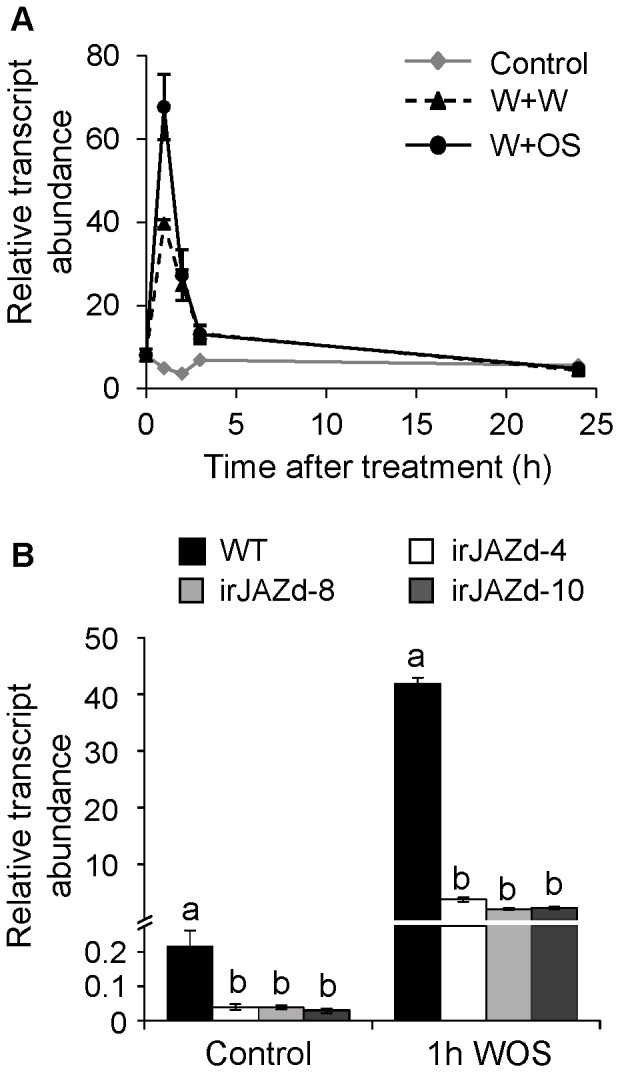
Regulation of *NaJAZd* transcripts and silencing efficiency in irJAZd plants. (A) *NaJAZd* transcript abundances ± SE were determined by quantitative real-time PCR (qPCR, *n = 3*) in samples from WT *N. attenuata* leaves treated with wounding and water (W+W), wounding and *M. sexta* oral secretions (W+OS) harvested at 0, 1, 2, 3, and 24 h after treatment (control leaves remained untreated). (B) Transcript abundances ± SE of *NaJAZd* determined in untreated (control) and 1 h W+OS-treated leaves of three independent inverted repeat (ir)-*NaJAZd*-silenced genotypes (irJAZd-4, -8, and -10) by qPCR (*n = 3*). Signals in A and B were normalized by housekeeping EF1α transcript abundances determined by qPCR in the same samples. Different letters in B indicate significant differences among the combination of identically treated genotypes (WT vs. independent *NaJAZd* silenced lines, irJAZd-4, -8, 10 by one-way-ANOVA (P≤0.05).

Previously, we reported 12 *JAZ* genes in *N. attenuata* [31], including the *NaJAZd* gene characterized in this study. First, we examined *NaJAZd* expression in the rosette leaves of *N. attenuata* plants after wound and water treatment (puncturing leaves with a fabric pattern wheel and supplying with 20 µL of water; W+W), simulated herbivore attack (wounds treated with 20 µL of 1∶10 diluted oral secretions isolated from specialist herbivore *Manduca sext*a (*M. sexta*) larvae; W+OS), and in untreated leaves by quantitative real-time PCR (qPCR). While both treatments strongly increased *NaJAZd* transcript accumulations compared to the levels in untreated leaves, W+OS-treatment dramatically amplified these increases (Figure 1A). The gene transcripts rapidly returned to basal levels within 3 h after treatment. To further explore the function of NaJAZd, we generated the inverted-repeat (ir) RNAi-mediated *NaJAZd*-silenced plants (irJAZd; Figure S1A and S1B) and selected the three best-silenced lines (irJAZd-4, -8, and -10; Figure 1B) for functional analysis. A single copy T-DNA insertion status of each line was confirmed by Southern blot analysis (Figure S1C).

While measuring the silencing efficiency of *NaJAZd* by RNAi, we also examined the expression of other *NaJAZ* genes (*NaJAZa*, -*b*, -*c*, -*e*, -*f*, -*h*, -*i*, -*j*, -*k*, -*l*, and -*m*) in irJAZd plants to identify possible cross-silencing effects and/or crosstalk among *JAZ* genes. Similar to our previous study with *NaJAZh*-silencing, which increased the expression of several other *JAZ* genes [31], we found a significantly higher expression of *NaJAZb* in both irJAZd examined lines (and higher expression of *NaJAZf*, -*j*, and -*m* in at least one line), suggesting a possible crosstalk or compensation responses in the JAZ regulatory network. In addition, we found a significant down-regulation of *NaJAZh* transcripts in response to W+OS-elicitation (Figure S2) in both examined lines, consistent with the result of nucleotide alignment of *NaJAZh* gene and irJAZd-silencing region which revealed a potential cross-silencing region of 20 nt (Text S1) between these sequences (no other identities ≥20 nt were found in any other known *N. attenuata JAZ* gene sequences). Recently, we analyzed in detail plants strongly silenced (>90%) in *NaJAZh* expression but the phenotypes found in irJAZh plants were not consistent with the irJAZd phenotypes described in the following chapters. For example, irJAZh plants showed significantly reduced nicotine levels and dramatic increases in TPI activity, as well as they developed spontaneous necrosis during aging [31], none of which could be found in irJAZd plants, suggesting that a partial silencing of NaJAZh was not sufficient to induce NaJAZh-associated phenotypes. We therefore proceeded with the search for specific NaJAZd silencing-associated phenotypes.

### NaJAZd-silencing Weakly Affects JA-dependent Defenses

**Figure 2:**
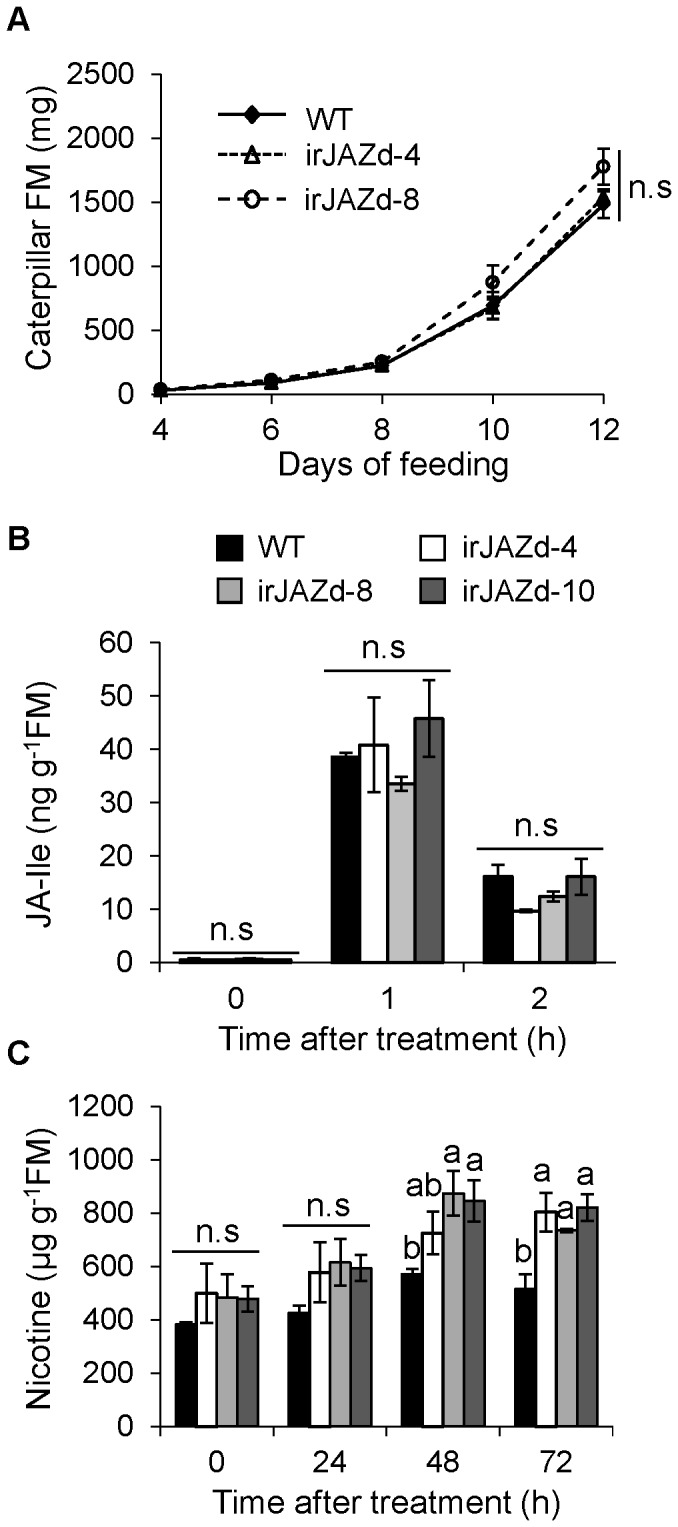
Defense responses against specialized herbivore *M. sexta* are mostly unaltered in irJAZd plants. (A) Herbivory performance of *M. sexta* on rosette leaves of WT and two independent irJAZd lines (irJAZd-4 and -8) was determined by measuring larval mass at 4, 6, 8, 10 and 12 d after placement of freshly hatched neonates on the plants. Mean fresh masses ± SE of irJAZd-4 and -8-fed caterpillars (*n = 20*) were not significantly different from WT-fed caterpillars. (B) Mean ± SE levels of JA-Ile (*n = 3*) determined by LC- ESI-MS/MS showed no significant differences in irJAZd compared to WT leaves. (C) Mean ± SE levels of nicotine (*n = 3*) determined by HPLC coupled to PDA (Photo Diode Array) detector were significantly higher at 48 and 72 h after W+OS treatment of irJAZd plants compared to WT. Statistical differences in (A)–(C) were determined by one-way-ANOVA (P≤0.05). Different letters indicate significant differences among the different genotypes (WT vs. independent NaJAZd silenced lines, irJAZd-4, -8, 10) at the same time points; n.s, not significantly different; FM, fresh mass.

To determine the role of NaJAZd in defense, we carried out performance assays with the specialist herbivore, *M. sexta,* with rosette stage WT and irJAZd plants. We placed a freshly hatched *M. sexta* neonate on the leaves of each 20 replicates of WT and irJAZd-4 and -8 plants and determined the mass of caterpillars after 4, 6, 8, 10, and 12 d of feeding on the plants (Figure 2A). *NaJAZd*-silencing did not affect the performance of *M. sexta* caterpillars as on both irJAZd genotypes and WT plants the larvae had similar growth rates.

To further test the hypothesis that NaJAZd is not a major player in defense against herbivores, we examined the levels of herbivore-induced phytohormones, JA-Ile (Figure 2B), jasmonic acid (JA), salicylic acid (SA) and abscisic acid (ABA; Figure S3) in rosette stage WT and irJAZd-4, -8, and -10 plants at 0, 1, and 2 h after W+OS treatment. Taking into account the data from three independently transformed irJAZd lines, the levels of JA-Ile (Figure 2B), and of other phytohormones (JA, SA and ABA; Figure S3) in W+OS-treated irJAZd plants were similar to those in WT at all examined time points, confirming that *NaJAZd*-silencing alone does not significantly alter the leaf levels of defense-related phytohormones, even though the transcript levels of *NaJAZd* were strongly elicited by W+OS in the leaves (Figure 1A). The basal levels of SA were slightly lower in all irJAZd lines; however, they rise to WT SA levels after W+OS treatment, suggesting a normal responsiveness and accumulation of SA in irJAZd plants during herbivory stress.

To gain additional insight in potential targets of NaJAZd, we analyzed several defense-related secondary metabolites after W+OS treatment. Nicotine is a well-known defense-related secondary metabolite in *Nicotiana* species [42], [43]. In contrast to the unaltered phytohormone levels, irJAZd leaves contained significantly more nicotine at 48 and 72 h after W+OS (Figure 2C), suggesting that NaJAZd may negatively contribute to biosynthesis of nicotine and/or suppress its transport from the roots. The accumulation of constitutive17-hydroxygeranyllinalool-diterpene glycosides (HGL-DTGs) [44], [45] was slightly higher in two irJAZd lines but not significantly different after W+OS treatment at 24–72 h compared to WT (Figure S4A). Trypsin protease inhibitors (TPIs) activity [46]–[48] in W+OS-treated irJAZd-4, -8, and -10 plants were not different from WT levels (Figure S4B). Apparently, higher amounts of nicotine in irJAZd compared to WT plants alone were not sufficient to alter the performance of a specialist herbivore (Figure 2A).

### NaJAZd-silencing does not Alter the Preferences of Native Herbivores in Nature

**Figure 3:**
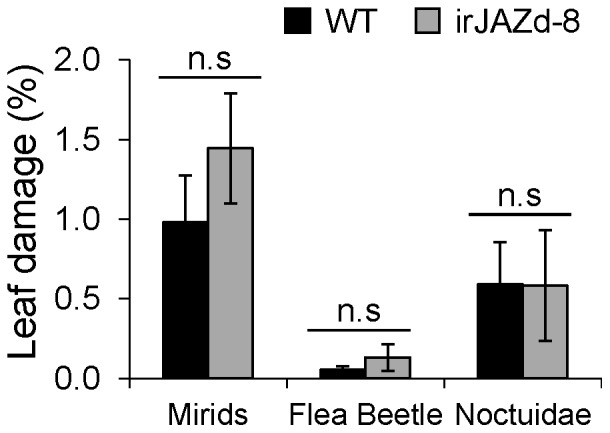
Plant damage caused by herbivores in *N. attenuata*’s native habitat. EV and irJAZd-8 plants were planted in a size-matched paired-design in their native habitat, Great Basin Desert, Utah, USA and damage by native herbivores was scored throughout the 2010 field season. Herbivore damage was determined as the % of leaf canopy damaged by (1) cell-damaging feeding of *Tupiocoris notatus* mirid bugs (mirids), (2) the small feeding holes that characterize flea beetle feeding, and (3) leaf chewing Lepidopteran larvae (Noctuidae). No significant differences (n.s) between the two genotypes determined by Student t-test were observed.

In natural environments, plants are exposed to substantially more stresses compared to their relatively safe containment in the glasshouse. We therefore examined if *NaJAZd*-silenced plants could perform differently in high stress conditions characterized by high UV irradiance, high and variable temperatures, low humidity and communities of voracious native herbivores. In the 2010 field season, we planted empty vector-transformed (EV) and irJAZd-8 plants in a pairwise design in the native habitat of *N. attenuata* (Great Basin Desert, Utah, USA) and compared herbivore damage to these plants (Figure 3). Field-grown irJAZd plants showed similar levels of damage from native herbivores, mirids (*Tupiocoris notatus*), flea beetles (*Epitrix* spp.), and noctuidae larvae (*Spodoptera* spp.) compared to WT plants, providing additional evidence that NaJAZd has only a minor role in defense against biotic and abiotic stresses. This prompted our intensive search for alternative functions of this protein.

### NaJAZd-deficiency Causes Increased Flower Abscission

**Figure 4:**
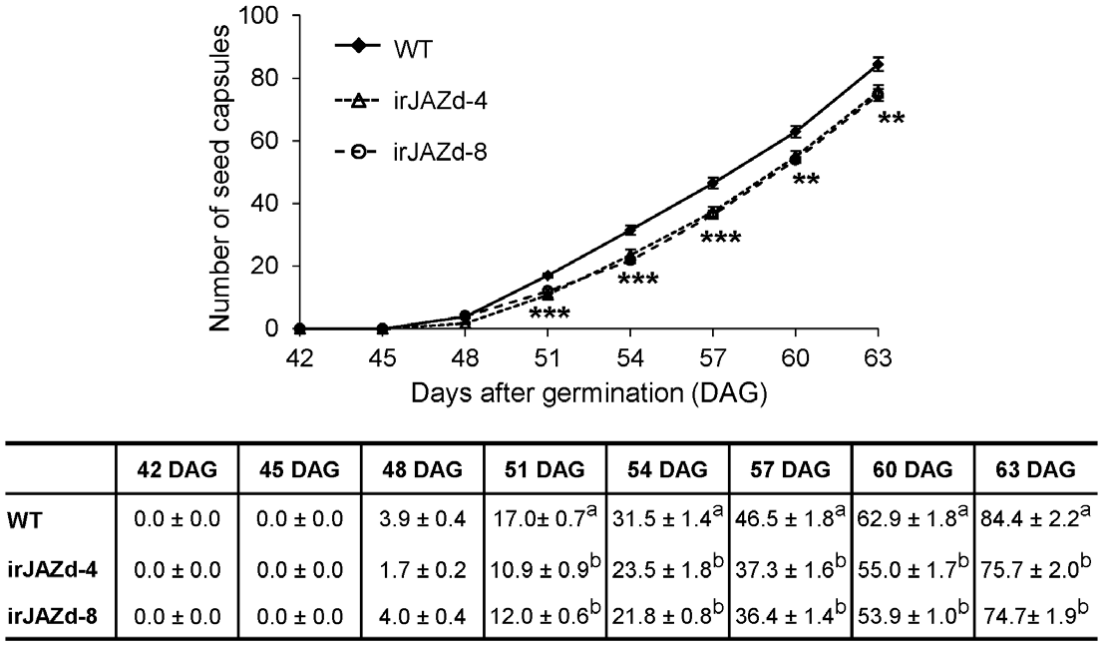
*NaJAZd*-silencing negatively affects seed capsule production. WT and two independent irJAZd lines (irJAZd-4 and -8) were grown in the glasshouse and their capsules were counted at specified time points. irJAZd plants produced significantly fewer seed capsules at 51, 54, 57, 60 and 63 d after germination compared to WT plants; significant differences between genotypes were determined at each time point by one-way-ANOVA (**P≤0.01, ***P≤0.001). There were no significant differences in number of seed capsules between two independent irJAZd lines (irJAZd-4 and -8). The exact numbers of capsules are displayed in table under the graph and different letters in the table indicate significant differences among the different genotypes (WT vs. independent NaJAZd silenced lines, irJAZd-4, -8) at the same time points. Differences at 42–48 d were not significant.

*NaJAZd*-silencing only slightly affected defense responses. Considering the extensively described role of JA in growth and development (reviewed in [9]), we decided to carefully examine the growth and development of irJAZd plants. The irJAZd plants showed no obvious vegetative growth deficiencies: they had similar size of rosettes, leaf shape, and stalk length (data not shown). However, in contrast to vegetative growth, their reproductive fitness was significantly compromised. During the harvesting of seeds, we noticed that irJAZd plants produced significantly less seed mass compared to WT plants. When we carefully counted the number of mature seed capsules during entire reproduction of WT and irJAZd-4 and -8 plants, both irJAZd lines had about 11∼35% fewer capsules compared to WT between 51–63 d after germination (Figure 4).

**Figure 5:**
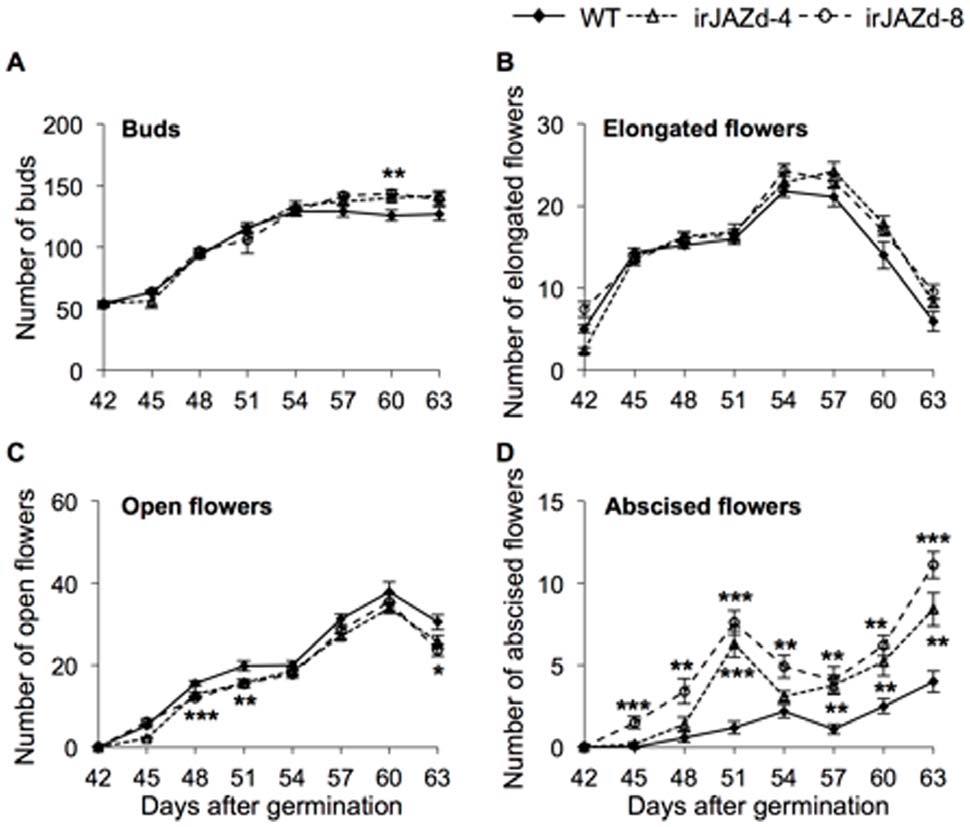
NaJAZd counteracts flower abscission in *N. attenuata*. Each individual plant was placed in an individual plastic tray (30×52 cm) in the glasshouse to avoid mixing of abscised flowers, and number of buds (A), elongated flowers (B), open flowers (C), and abscised flowers (D) in each plant from 42 d to 63 d after germination was determined in 3 d intervals. Both irJAZd-4 and -8 plants had similar number of buds and elongated flowers as WT plants but displayed significantly reduced numbers of open flowers (significant at 48, 51, 63 d) and higher numbers of abscised flowers (significant at most time points) compared to WT plants. Significant differences between genotypes were determined separately for each time point by one-way-ANOVA (*P≤0.05, **P≤0.01, ***P≤0.001).

We hypothesized that NaJAZd was involved in flower initiation, which would ultimately affect the lifetime seed capsule production in irJAZd plants. However, the numbers of flower buds in irJAZd plants seemed comparable in WT and irJAZd plants and flower parameters such as degree of flower opening, pollen maturation or length of pistils were not visibly altered in irJAZd plants. In addition, we examined if self-pollination ability was impaired in irJAZd flowers by hand pollination experiments using ripe pollen from the same flowers and spreading it on stigma with fine brush (Figure S5). The hand pollination, assuring that each stigma received sufficient amount of pollen in a timely coordinated fashion, failed to recover the formation of seed capsules in irJAZd plants to WT levels. These results suggested that irJAZd flowers have normal anthesis and otherwise completely normal morphology (Figure S6) but experience another problem in flower development. We therefore conducted another more detailed experiment in which we quantified flower production distinguishing 4 categories: buds, elongated flowers, fully opened flowers and abscised flowers, which were counted every 3 d starting 42 d after germination when the first buds and a few elongated flowers but no open flowers were present on the plants (Figure 5). To prevent mixing of abscised flowers from different plants, we placed each single plant in individual 30×52 cm plastic tray which captured all abscised flowers from a single plant. While irJAZd plants had similar or even higher number of buds and elongated flowers, they produced significantly fewer open flowers on 48, 51, and 63 d-old plants and correspondingly higher numbers of abscised flowers at these and additional time points. Notably, the abscised flowers were all fully open flowers; abscission of younger stages or flower buds was not occurring. These data suggested that the function of NaJAZd is to prevent flower abscission in the later stages of flower development that directly affects lifetime production of seed capsules and fitness of *N. attenuata*. Whether this was mediated by direct function of NaJAZd in flowers was examined next.

### Phytohormones and Gene Expression in N. attenuata Flowers

**Figure 6:**
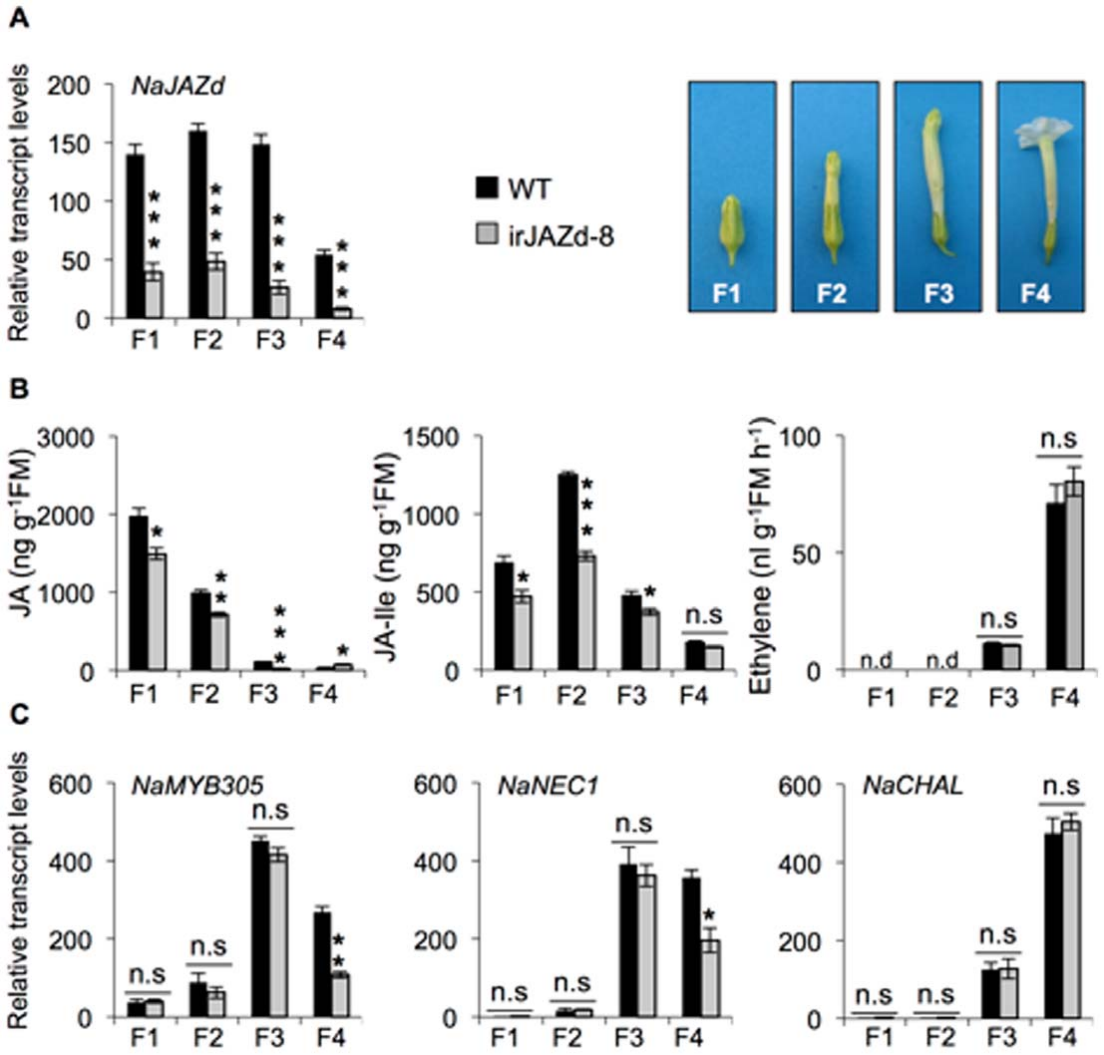
NaJAZd regulates phytohormone levels and flower development-related genes. WT and irJAZd plants (irJAZd-8) were grown in the glasshouse and four different developmental stages of flowers (F1, F2, F3, and F4) were collected 57 d after germination. (A) Transcript abundances of *NaJAZd* determined by qPCR in irJAZd-8 flowers were significantly lower compared to WT. (B) Mean JA and JA-Ile levels ± SE determined by LC-ESI-MS/MS using individual stage flowers and mean ethylene levels ± SE measured by photoacoustic spectrometer using a mixture of five flowers of each stage. (C) Transcripts abundances ± SE of flower development-related genes, *NaMYB305*, *NaNEC1*, and *NaCHAL* determined by qPCR: irJAZd-8 plants were impaired in expression of *NaMYB305* and *NaNEC1*genes in last stage of flower development (F4) while *NaCHAL* transcripts remained unaltered in irJAZd-8 compared to WT flowers. Bars ± SE in (C) show *EF1α*-normalized relative transcript abundances. Statistical differences in phytohormones, JA, JA-Ile, ethylene (*n = 4*) and transcript abundances (*n = 4*) were determined by Student t-test. Asterisks represent significant differences between WT and irJAZd in same stage of flowers (*P≤0.05, **P≤0.01, ***P≤0.001); n.s, not significantly different; FM, fresh mass.

To elucidate the molecular mechanisms involved in NaJAZd-regulated flower abscission, we analyzed the levels of phytohormones and flower-related gene expression at four different developmental stages of flowers in WT and irJAZd-8 plants: buds (F1), early elongated flowers (∼10 mm length, F2), fully elongated flowers (still green and completely closed corollas, F3) and open flowers (completely opened white corollas, F4). First, we determined the expression of *NaJAZd* in WT and irJAZd-8 flowers to examine if (1) *NaJAZd* is expressed in stage-specific manner, and (2) to evaluate the efficiency of gene silencing in irJAZd flowers by RNAi. In WT plants, the gene showed comparably high transcript levels during F1–F3 stages but its expression declined in the F4 stage. *NaJAZd* transcript levels were strongly reduced in irJAZd-8 flowers compared to WT levels (Figure 6A).

Ethylene is known to be one of the important signals controlling flower abscission in plants (reviewed in [49]). The analysis of nearly 300 plant species showed that flower abscission in plants is highly sensitive to ethylene [50]. We therefore investigated the levels of ethylene and its possible role in enhanced flower abscission in irJAZd-8 plants. Ethylene emissions increased in a stage dependent manner; however, we found no significant differences between WT and irJAZd-8 flowers at all examined developmental stages (Figure 6B). These data suggest that enhanced flower abscission in irJAZd plants is independent of ethylene concentrations in irJAZd-8 flowers.

Because JA is also known to regulate flower development in plants, we analyzed JA and JA-Ile levels using entire homogenized flowers. Interestingly, at three developmental stages (F1, F2, and F3) irJAZd-8 flowers had significantly reduced levels of JA and JA-Ile compared to WT flowers (Figure 6B). It suggested that NaJAZd might be regulating flower abscission process via the regulation of JA and JA-Ile levels and/or JA-Ile-mediated signaling process. How a putative negative regulator NaJAZd contributes to the accumulation of JA remains to be elucidated.

Finally, we examined the expression of several flower development-related genes. The R2R3-MYB transcription factors are known to regulate stamen maturation, flower opening and nectar production (reviewed in [51]). Recently, the function of *MYB305* gene in controlling flower opening and floral nectar production in petunia, *N. tabacum* and *N. attenuata* was reported [52], [53]. The *N. attenuata* plants strongly silenced in the expression of *MYB305* showed premature flower abscission in early flower developmental stages: their flowers failed to enter anthesis and eventually, the plants did not produce any seed capsules. Although irJAZd flowers did not show anthesis-related phenotypes as described in the previous section, premature flower abscission phenotype strongly resembled those of irMYB305 plants but the abscission was shifted to later stages in flower development. To examine a possible relationship between NaMYB305 and NaJAZd, we analyzed *NaMYB305* expression at four different stages of WT and irJAZd-8 flowers (Figure 6C). In both WT and irJAZd-8 flowers, *NaMYB305* expression gradually increased from F1 to F3, corroborating previous studies [52]. However, the irJAZd-8 flowers contained significantly fewer *NaMYB305* transcripts than did F4 stage WT flowers, suggesting that NaJAZd might be required for maintaining the appropriate levels of *NaMYB305* in open stage flowers. Because fully silenced irMYB305 plants lost all their flowers, it is likely that moderate reductions in *NaMYB305* levels observed in this study could be responsible for the abscission of a certain portion of flowers in irJAZd plants.

To further examine the NaMYB305 deficiency, we analyzed the expression of *NaNEC1* (nectarine 1) and *NaCHAL* (chalcone synthase) genes (Figure 6C) which are located downstream of MYB305 regulator in petunia and tobacco [52], [54], [55]. Consistent with *NaMYB305* expression, *NaNEC1* was similarly down regulated in F4 stage flowers in irJAZd-8 plants compared to WT flowers. However, *NaCHAL* expression was not significantly different between WT and irJAZd-8 flowers (Figure 6C), showing an expression pattern which closely tracked flower ethylene emissions (Figure 6B). In an independent experiment, we also analyzed in detail the hormone contents and gene transcript levels in irJAZd-4 and WT flowers (Figure S7). Identical changes in JA, JA-Ile contents and *NaMYB305* transcript accumulation were observed but the changes were less pronounced compared to the independently transformed line irJAZd-8, which was consistent with the lower number of abscised flowers found in irJAZd-4 line (Fig. 5D). In irJAZd-4 line, the levels of *NaNEC1* transcripts were not yet reduced, showing a time delay between accumulation of the MYB305 regulator and the expression of downstream-regulated gene, *NEC1*.

### Global Transcriptional Changes Associated with NaJAZd-silencing in N. attenuata Leaves

Because *NaJAZd* gene was strongly induced by W+OS treatment in the leaves, we conducted an additional microarray experiment focused on global leaf gene expression 2 h after W+OS-treatment. *NaJAZd*-silencing down-regulated a large number of genes (10321 of 43504 microarray probes) but it up-regulated only a relatively small number (38) of genes. The list of more than 3 times up (20)- and down (99)-regulated genes in irJAZd compared to WT plants was annotated and categorized according to established GO categories (Tables S1 and S2). Interestingly, several primary metabolic genes, such as sugar transporter SWEET3 (4.93-fold), unknown glycosyltransferase (4.17-fold), fructokinase (3.76-fold), putative beta-1, 3-glucan synthase (3.76-fold) and 6-phosphofructokinase 4 (3.7-fold) were strongly down-regulated in irJAZd leaves compared to WT leaves. These results suggest that, apart from direct changes in flowers caused by *NaJAZd*-silencing, the enhanced flower abscission phenotype in irJAZd plants could be due to a reduced nutrient availability in the flowers as leaves are providers of all essential nutrients required for successful flower development. Previous studies suggested that JA signaling might regulate sink-source relationship by regulating expression and/or accumulation of vegetative storage proteins (VSPs) in soybean plants [56], [57]. Further experiments are required to elucidate the emerging pleiotropic roles of NaJAZd in plant metabolism, development and defense.

## Discussion

### NaJAZd is a Minor Defense Regulator in *N. attenuata*

Previously, a dominant-negative truncated forms of NtJAZ1and NtJAZ3 proteins from *N. tabacum*, a close homologues of *N. attenuata* NaJAZd and NaJAZa, respectively, repressed the MeJA-induced nicotine and related alkaloid accumulations in cultivated tobacco cells [35]. However, truncation of JAZ proteins affects the overall JAZ-mediated signaling so the plants become completely “deaf” to JA signaling. In other words, experiments with truncated JAZs can only tell us that certain metabolites, such as nicotine, are indeed JAZ-regulated but cannot pinpoint the causative JAZ protein(s) involved. In contrast, targeted gene silencing is more useful but such analyses are frequently confounded by redundancy of gene function, and/or the lack of sophisticated, ecologically realistic phenotypic screens. Despite predicted and/or observed redundancy in the function of JAZ proteins [16], [17], [58], we reported that NaJAZh alone is able to suppress the accumulation of two herbivore-induced defense metabolites, HGL-DTGs and TPIs in *N. attenuata*. In addition, silencing of *NaJAZh* by RNAi strongly reduced the performance of *M. sexta* larvae on these plants [31]. In the follow up experiments, we therefore decided to use gene silencing to examine the function of NaJAZd.

Overall, our data suggest that NaJAZd protein is another negative JAZ regulator involved in defense, particularly in nicotine accumulation. *NaJAZd*-silencing allowed higher accumulation of nicotine in simulated herbivory-treated plants at 48 and 72 h (Figure 2C). Regulation of nicotine levels by NaJAZd was specific to this alkaloid as other defensive secondary metabolites such as HGL-DTGs or TPIs, previously shown to be controlled by NaJAZh [31], were not altered. However, the control of NaJAZd over nicotine levels was marginal as irJAZd plants did not contain constitutively increased nicotine levels as would be expected if NaJAZd was a master repressor of nicotine biosynthesis. Previously, silencing of NaJAZh, a master repressor of HGL-DTGs and TPIs caused significant increase in basal levels of these otherwise inducible metabolites in irJAZh plants [31]. Eventually, the changes in nicotine levels in *NaJAZd*-silenced plants were not sufficient to affect growth of a specialist herbivore *M. sexta* feeding on irJAZd plants in glasshouse (Figure 2A), and several native herbivores of *N. attenuata* in native habitat of this plant (Figure 3).

Our initial data thus suggested that NaJAZd might not be a major player in defense. We therefore searched for alternative functions of this protein, finding an independent, fitness-related role of NaJAZd. The expression of *NaJAZd* was required for WT-level retention of flowers in *N. attenuata* inflorescences, a finding not surprising as JA is known to control various aspects of flower development. Furthermore, functional specialization of JAZ proteins in both defense and growth has already been proposed by other authors [10], [30], [31].

### NaJAZd Affects JA Signaling in Flowers and Counteracts Flower Abscission

The irJAZd plants were not different from WT in their vegetative growth; however, more irJAZd flowers abscised compared to WT, which significantly reduced the number of mature seed capsules (Figures 4 and 5). Ethylene is known to be a critical regulator of flower abscission (reviewed in [49]), but in follow up analyses, ethylene emissions were found unaltered in irJAZd flowers compared to WT (Figure 6B). Plants blocked in ethylene perception (*etr1* mutants) show a typical flower corolla-retention phenotype where corollas remain attached to even ripe capsules [59]–[61], demonstrating that perception of the post-pollination ethylene burst triggers corolla abscission after successful pollination [61]–[63]. However, in irJAZd plants, whole flowers abscised after separation of pedicels from inflorescences, which was a distinctly different process from that of the ethylene-mediated corolla abscission.

In contrast to ethylene, the patterns of JA and JA-Ile accumulation are altered in irJAZd flowers compared to WT (Figure 6B), which re-connects NaJAZd to its expected function as an endogenous regulator of JA signaling, albeit in flowers. It has been widely reported that JA affects flower development [23], [64], [65], but JA function has been typically associated with male sterility. For example, defects in pollen maturation and pistil elongation disabled efficient self-pollination in COI1 mutant plants [14], [66], [67]). Here, our data provide a novel insight into JA function in flower development. irJAZd phenotype is likely a combined effect of reduced JA and JA-Ile levels and/or impaired JA signaling due to silencing of NaJAZd repressor in the flowers. At present, no other JA-deficient *N. attenuata* genotypes, including irAOC (strongly silenced in expression of allene oxide cyclase) and irCOI1 (silenced in expression of coronatine insensitive 1) plants have been reported to show a similar flower abscission phenotype. Whether the effect of NaJAZd is on the enzymes that degrade JA or promotes JA biosynthesis in the flowers by suppressing a putative negative regulator of biosynthetic genes, remains to be determined. From our data and the expression of the key flower regulator NaMYB305, we propose that the function of NaJAZd is to maintain optimal levels of JA throughout flower development, which in turn, provides sufficient expression and function of MYB305 transcriptional regulator. Previously, plants silenced in expression of *NaMYB305* gene were completely sterile due to complete abscission of buds and early elongated flowers [52]. The silencing of *NaMYB305* in *N. attenuata* was partially counteracted by inhibiting ethylene perception with 1-MCP treatments, and it is therefore possible that the lack of NaJAZd and dysfunction of MYB305 may be caused by an exaggerated sensitivity to otherwise normal levels of ethylene in irJAZd flowers.

The homologues of NaMYB305 in petunia and *N. tabacum* regulate flower-specific flavonoid biosynthetic genes (phenylalanine ammonia-lyase; *PAL*, chalcone isomerase; *CHI*, and chalcone synthase; *CHS*) and two nectarines (nectarin1; *NEC1*, nectrain5; *NEC5*). Nectarines, in particular, are known to be involved in direct flower defense, which may link NaJAZd function back to defense. Previously, NEC1 has been shown to control the production of hydrogen peroxide (H_2_O_2_) in nectar together with NEC3 and NEC 5 proteins and high levels of antimicrobial H_2_O_2_ (up to 4 mM) are thought to protect the gynoecium and developing ovules from invading microorganisms [55], [68], [69]. Recently, MYB305 has been also shown to mediate additional functions in the maturation of the tobacco nectary by controlling the expression of several starch metabolic genes [53].

Although we found altered JA levels and direct changes in flower gene expression, it should not be forgotten that NaJAZd is strongly regulated by herbivory stress in *N. attenuata* leaves. The regulatory role of NaJAZd over several primary metabolic genes in leaves during simulated herbivory, as revealed by microarray analysis of the leaves, offers an alternative mode of action for NaJAZd via control and/or redistribution of nutrients, which then might indirectly affect flower and capsule development in *N. attenuata*.

### Conclusions

Increased flower abscission in *NaJAZd*-silenced plants points to a novel function of JAZ proteins in plants. The absence of NaJAZd negatively affected the fitness of plants as the production of seed capsules (and seeds) in irJAZd plants were reduced by around 20 percent. Our data suggest that NaJAZd is required for a proper accumulation and/or maintenance of *NaMYB305* transcript levels in developing flowers, revealing a new function and requirement of *NaMYB305* in flower retention during later stages of flowering that can optimize fitness and seed production in plants.

## Materials and Methods

### Plant material and Growth Conditions

All experiments were conducted with 31^st^ inbred generation of *N. attenuata*. Seeds were germinated and grown in the glasshouse as previously described in Krügel et al. [70]. Plants were maintained under 16 h daylight supplemented by Philips Master Sun-T PIA Agro 400 W or 600 W sodium lights at 23–25°C and 8 h dark at 19–23°C, 45 to 55% relative humidity.

To generate inverted repeat (ir) JAZd plants, we cloned a 303 bp fragment of *NaJAZd* gene (Figure S1A) as an inverted repeat into pSOL8 transformation vector [71] containing hygromycin (*hptII*) resistance gene as plant selection maker (Figure S1B). *Agrobacterium tumefaciens*-mediated plant transformation was conducted essentially as described in Krügel et al. [70]. The best *NaJAZd*-silenced, single T-DNA insertion transgenic lines (irJAZd-4, -8, and -10) were selected on hygromycin and subjected to Southern blot (Figure S1C) and quantitative real-time PCR (qPCR; Figure 1B)) analyses as described in Oh et al. [31].

Experiments were conducted with transition leaves (i.e., leaves undergoing the source-sink transition at node -1) using approximately 30-d-old rosette-stage *N. attenuata* plants. Four different developmental stages of flowers were collected from approximately 57-d-old flowering *N. attenuata* plants.

### Quantitative Real-time PCR

Total RNA was extracted from approximately 100 mg of frozen leaves or flower tissues ground in liquid nitrogen using Trizol reagent as recommended by manufacturer (Invitrogen). Total RNA was treated with RQ1 RNase-Free DNase (Promega), phenol extracted and precipitated by addition of 3M sodium acetate (pH 5.2) and 100% ethanol. First strand cDNA was synthesized from 1 µg of RNA using oligo-dT primer (Fermentas) and RevertAid™ H Minus reverse transcriptase (Fermentas) following manufacturer’s protocol. Quantitative real-time PCR (qPCR) was conducted with the core reagent kit for SYBR Green I (Eurogentec) and gene-specific primer pairs (Table S3) using Mx3005P PCR cycler (Stratagene). Relative transcript abundances were calculated from dilution series of standard curves and normalized by *NtEF1α* gene (*N. tabacum* elongation factor 1*α*) expression.

### Herbivore Performance in the Glasshouse

To determine herbivore performance, freshly hatched specialist herbivore *M. sexta* neonates were placed on selected rosette leaves of 20 each WT and two independent irJAZd line plants (irJAZd-4 and -8). The larval fresh mass was measured on 4^th^, 6^th^, 8^th^, 10^th^, 12^th^ d after initial feeding.

### Phytohormone Analyses

To determine JA, JA-Ile, SA and ABA levels in WT and irJAZd plants, phytohormones were extracted from approximately 100 mg frozen leaves or flowers. Plant tissues were homogenized with 1 mL of internal standard (200 ng of [^2^H_2_]JA, and 40 ng each of JA-[^13^C_6_]Ile, [^2^H_4_]SA and [^2^H_6_]ABA)-spiked ethyl acetate and 2 steel balls in a Genogrider 2000 (SPEX Certi Prep) at 1000 strokes per minute for 10 min. The extracts were centrifuged at 16,100 *g* at 4°C for 15 min, and the upper organic phases were transferred to clean microcentrifuge tubes and dried in vacuum concentrator (Eppendorf) at 30°C. The residues were re-suspended in 500 µL (for leaf) or 200 µL (for flowers) of 70% (v/v) methanol : water and centrifuged. 10 µL of particle free supernatant was analyzed in Varian 1200 LC-ESI-MS/MS system (Varian) as described in Oh et al. [31].

Ethylene emissions were measured with a photoacoustic spectrometer (INVIVO; https://www.invivo-gmbh.de) as described in von Dahl et al. [72]. irJAZd and WT plants were grown in the glasshouse until flowering stage and five flowers were collected from each stage of flowers to measure ethylene in 250 mL flasks. Flowers were incubated for 5 h to accumulate ethylene in the flasks and accumulated ethylene in the headspace was flushed with a 130 to 150 mL/min flow of purified air into spectrometer where it was measured against known amount of ethylene standard. The results were normalized by fresh mass of flowers used in each measurement.

### Analysis of Secondary Metabolites by HPLC

Plants materials (∼100 mg) were homogenized with 1 mL of acetate buffer (60% buffer A; 2.3 mL/L of acetic acid, 3.41 g/L ammonium acetate adjusted to pH 4.8 with 1 M NH_4_OH, and 40% (v/v) methanol) and analyzed by HPLC (Agilent-HPLC 1100 series) coupled with PDA (Photo Diode Array, Agilent) and ELS (Evaporative Light Scattering, Varian) detectors as described in Oh et al. [31].

### Field Bioassays

The field experiments were performed in the native habitat of *N. attenuata*, the Lytle Ranch Preserve, Utah, Santa Clara, USA. The release of transgenic plants was carried under the Animal and Plant Health Inspection Service (APHIS) notification 06-242-3r-a3 and the seeds were imported to USA under permit number 07-341-101n. The seeds of EV and irJAZd-8 plants were germinated on Gamborg’s B5 medium as described in earlier section (Plant material and growth conditions). About 15 d-old seedlings were transferred to pre-hydrated 50 mm peat pellets (Jiffy 703, http://www.jiffypot.com) and seedlings were gradually adapted to the high light and low relative humidity of the habitat over a 2-week-period. Finally, pre-adapted rosette-stage plants were transplanted on the field plot and watered daily until roots have established for approximately 2 weeks, after which the plants were grown without watering. 15 pairs of EV and irJAZd-8 plants were planted and grown in the field plot and monitored for damage from native herbivores. Damage of plants by native herbivores was determined by estimating the percentages of total leaf area of plants damaged by each herbivore: Noctuidae larvae, *Spodoptera* spp.; flea beetles, *Epitrix* species; and mirids, *Tupiocoris notatus*. A result of representative measurement conducted on 15^th^, May, 2010 is shown in the Figure 3.

### Seed Capsule and Flower Counts

The number of seed capsules at four different developmental stages of flowers (buds, elongated-, open-, and abscised flowers) were counted in 3 d intervals from 42 d until 63 d after germination that covered complete reproductive stage of *N. attenuata* plants. Seed capsules were counted after complete maturation of plants. For counting abscised flowers, the plants were placed in separate plastic trays (30×52 cm) and kept apart to avoid mixing of abscised flowers. Every 3 d, buds, elongated- and open flowers on the plants, and newly abscised flowers on each tray were counted.

### Hand-pollination Experiments

Plants were kept in the glasshouse until flowering stage (approximately 55 d after germination) and all fully elongated but still green flowers of each WT, irJAZd-4 and irJAZd-8 plants were labeled previous evening and half of the flowers were hand-pollinated when the flowers opened next morning. Control half-set of the plants remained intact and was allowed to self-pollinate only. Hand-pollinations were repeated 4-times with ripe pollen from the same flowers by spreading pollen on stigma with a fine brush and the percentage of mature capsules resulting from hand- and self-pollinated flowers were counted after 10 d period.

### Microarray Experiment

Untreated and W+OS-treated leaves of 30-d-old WT and irJAZd-8 plants were used for microarrays. Total RNA was extracted as described in Kistner and Matamoros, [73] and cDNA preparation and hybridizations were performed as described in Kallenbach et al. [74]. Raw microarray data were normalized by 75 percentile, log2 transformed and processed by SAM software version 3.11 (Significance Analysis of Microarrays; Stanford University, USA; [75]). For selection and annotation of genes, false discovery rates (FDR) ≤2.09% and greater than 3-fold signal changes (irJAZd vs. WT) were used. QC table showing the technical variability of 30 selected control genes spotted in 10 different locations of microarray chip is shown in Table S4. All data were deposited to public GEO (Gene Expression Omnibus) repository under accession number GSE43395. The genes were annotated after processing each entry by Blast-X program (E-value <1–5e) and classified into groups based on GO classification from TAIR (http://www.arabidopsis.org).

### Statistical Analyses

Data were analyzed with StatView 5.0 software (SAS institute) using appropriate methods such as Student t-test for pair comparisons and ANOVA Fisher’s PLSD for multiple samples.

## Supporting Information

Figure S1 **Generation of stable** ***NaJAZd*** **-silenced** ***N. attenuata*** **plants.** (A) A 303 bp region in *NaJAZd* gene used for gene silencing is shown in red letters. (B) The pSOL8JAZd vector containing inverted repeat of *NaJAZd* gene used for *Agrobacterium tumefaciens*-mediated transformation and generation of stably silenced *N. attenuata* irJAZd plants. (C) Southern blot analysis of 6 independently transformed irJAZd (irJAZd-1, -2, -4, -8, -9, and -10) lines and WT. The genomic DNA was digested with *Xba*I enzyme and hybridized with a ^32^P-labeled probe coding for the hygromycin resistance gene located between the right and left T-DNA borders of the transformation vector pSOL8JAZd. The black boxes indicate single T-DNA insertion lines selected for further experiments: irJAZd-4, -8, and -10.
(TIF)

Figure S2 **Transcript abundances of other** ***NaJAZ*** **genes in irJAZd plants.** Transcript abundances of other *NaJAZ* genes were determined by qPCR in the leaves of irJAZd and WT plants before and 1 h after W+OS elicitation; bars indicate *EF1α*-normalized relative transcript abundances ± SE (*n = 3*) and different letters indicate significant differences among the combination of genotypes (WT vs. independent *NaJAZd*-silenced lines, irJAZd-8, 10) and treatments determined by one-way-ANOVA (P≤0.05); n.s, not significantly different.
(TIF)

Figure S3 ***NaJAZd*** **-silencing does not significantly alter basal or herbivory-induced phytohormones levels.** Rosette stage plants of WT and irJAZd (irJAZd-4, -8 and -10) were treated with W+OS and harvested before, 1 and 2 h after treatment. Mean ± SE levels of JA, ABA and SA (*n = 3*) were determined by LC- ESI-MS/MS using internal deuterium-labeled phytohormone standards. Different letters indicate significant differences among the different genotypes (WT vs. independent *NaJAZd* silenced lines, irJAZd-4, -8, 10) at the same time points by ANOVA (P≤0.05); n.s, not significantly different. FM, fresh mass.
(TIF)

Figure S4 ***NaJAZd*** **-deficiency does not affect levels of defense-related secondary metabolites, HGL-DTGs and TPIs, in irJAZd plants.** Rosette stage WT and irJAZd (irJAZh-4, -8 and -10) plants were treated with W+OS and harvested before and 24, 48, and 72 h after treatment for determination of total HGL-DTGs levels and trypsin protease inhibitors (TPIs) activity. (A) Mean ± SE levels of total HGL-DTGs measured by HPLC coupled to ELS (Evaporative Light Scattering) detector (*n = 3*). (B) Mean ± SE levels of TPI activities determined by radial diffusion assay (*n = 3*). Different letters in A and B indicate significant differences among the different genotypes (WT vs. independent *NaJAZd* silenced lines, irJAZd-4, -8, 10) at the same time point determined by one-way-ANOVA (P≤0.05); n.s, not significantly different. FM, fresh mass.
(TIF)

Figure S5 **Hand-pollination does not rescue seed capsule formation in irJAZd plants.** Plants were kept in the glasshouse until flowering stage (approximately 55 d after germination) and, in the previous evening, all fully elongated flowers ready to open next morning were labeled with color strings. In half of the plants, hand- pollination was conducted while second half remained exclusively self-pollinated. Approximately 10 d later, mature seed capsules resulting from labeled flowers in each group were counted and percentage of capsules originating from self- and hand-pollination groups of WT and irJAZd plants were determined (*n = 24*). Different letters indicate significant differences among the different genotypes (WT vs. independent *NaJAZd*-silenced lines, irJAZd-4, -8) at the same condition determined by one-way-ANOVA (P≤0.05).
(TIF)

Figure S6 **Flowers and buds of irJAZd transgenic and WT plants at F1–F4 stages of development.** Flower buds and flowers were detached from 57-d-old plants and photographed to capture the highly similar morphology of flowers in two transgenic irJAZd-4 and -8 lines and WT.
(TIF)

Figure S7 **NaJAZd regulates phytohormone levels and flower development-related genes.** WT and irJAZd-4 plants were grown in glasshouse and four different developmental stages of flowers (F1, F2, F3, and F4) were collected 57 d after germination. (A) Transcript abundances of *NaJAZd* determined by qPCR in irJAZd-4 flowers were significantly lower compared to WT. (B) Mean JA and JA-Ile levels ± SE determined by LC-ESI-MS/MS using four individual stage flowers and mean ethylene levels ± SE measured by photoacoustic spectrometer using a mixture of five flowers of each stage. (C) Transcripts abundances of flower development-related genes, *NaMYB305*, *NaNEC1*, and *NaCHAL* determined by qPCR: irJAZd-4 plants were impaired in expression of *NaMYB305* gene in last stage of flower development (F4) while *NaNEC1* and *NaCHAL* transcripts were unaltered in irJAZd-4 compared to WT flowers. Bars ± SE in (C) show *EF1α*-normalized relative transcript abundances. Statistical differences in phytohormones, JA, JA-Ile, ethylene (*n = 4*), and transcript abundances (*n = 4*) were determined by Student t-test. Asterisks represent significant differences between WT and irJAZd in same stage of flowers (*P≤0.05, **P≤0.01, ***P≤0.001); n.s, not significantly different; FM, fresh mass.
(TIFF)

Table S1 **Up-regulated genes in irJAZd plants compared to WT plants determined by microarrays.**
(PDF)

Table S2 **Down-regulated genes in irJAZd plants compared to WT plants determined by microarrays.**
(PDF)

Table S3 **Primer sequences used in quantitative real time PCR (qPCR).**
(PDF)

Table S4 **QC table showing technical variability of 30 selected control genes spotted in 10 different locations on microarray chips.**
(PDF)

Text S1 **Nucleotide sequence alignment of** ***NaJAZ*** **genes and inverted repeat (ir) construct used for NaJAZd silencing in irJAZh plants.**
(PDF)

## References

[1] Strauss SY, Rudgers JA, Lau JA, Irwin RE (2002) Direct and ecological costs of resistance to herbivory. Trends Ecol Evol 17: 278–285.

[2] Kessler A, Baldwin IT (2004) Herbivore-induced plant vaccination. Part I. The orchestration of plant defenses in nature and their fitness consequences in the wild tobacco *Nicotiana attenuata* . Plant J 38: 639–649. 15125770 10.1111/j.1365-313X.2004.02076.x

[3] Zavala JA, Patankar AG, Gase K, Baldwin IT (2004) Constitutive and inducible trypsin proteinase inhibitor production incurs large fitness costs in *Nicotiana attenuata* . Proc Natl Acad Sci USA 101: 1607–1612. 14757829 10.1073/pnas.0305096101 PMC341788

[4] Steppuhn A, Baldwin IT (2008) Induced defenses and the cost-benefit paradigm. In: Schaller A, editor: Springer Netherlands. 61–83.

[5] Baena-González E, Sheen J (2008) Convergent energy and stress signaling. Trends Plant Sci 13: 474–482. 18701338 10.1016/j.tplants.2008.06.006 PMC3075853

[6] McSteen P, Zhao Y (2008) Plant hormones and signaling: common themes and new developments. Dev Cell 14: 467–473. 18410724 10.1016/j.devcel.2008.03.013

[7] Balbi V, Devoto A (2008) Jasmonate signalling network in *Arabidopsis thaliana*: crucial regulatory nodes and new physiological scenarios. New Phytol 177: 301–318. 18042205 10.1111/j.1469-8137.2007.02292.x

[8] Schaller A, Stintzi A (2009) Enzymes in jasmonate biosynthesis-structure, function, regulation. Phytochemistry 70: 1532–1538. 19703696 10.1016/j.phytochem.2009.07.032

[9] Wasternack C (2007) Jasmonates: an update on biosynthesis, signal transduction and action in plant stress response, growth and development. Ann Bot 100: 681–697. 17513307 10.1093/aob/mcm079 PMC2749622

[10] Howe GA, Jander G (2008) Plant immunity to insect herbivores. Annu Rev Plant Biol 59: 41–66. 18031220 10.1146/annurev.arplant.59.032607.092825

[11] Wu J, Baldwin IT (2010) New insights into plant responses to the attack from insect herbivores. Annu Rev Genet 44: 1–24. 20649414 10.1146/annurev-genet-102209-163500

[12] De Geyter N, Gholami A, Goormachtig S, Goossens A (2012) Transcriptional machineries in jasmonate-elicited plant secondary metabolism. Trends Plant Sci 17: 349–359. 22459758 10.1016/j.tplants.2012.03.001

[13] Gutierrez L, Mongelard G, Floková K, Păcurar DI, Novák O, et al (2012) Auxin controls *Arabidopsis* adventitious root initiation by regulating jasmonic acid homeostasis. Plant Cell 24: 2515–2527. 22730403 10.1105/tpc.112.099119 PMC3406919

[14] Feys B, Benedetti CE, Penfold CN, Turner JG (1994) *Arabidopsis* mutants selected for resistance to the phytotoxin coronatine are male sterile, insensitive to methyl jasmonate, and resistant to a bacterial pathogen. Plant Cell 6: 751–759. 12244256 10.1105/tpc.6.5.751 PMC160473

[15] Devoto A, Ellis C, Magusin A, Chang HS, Chilcott C, et al (2005) Expression profiling reveals COI1 to be a key regulator of genes involved in wound- and methyl jasmonate-induced secondary metabolism, defence, and hormone interactions. Plant Mol Biol 58: 497–513. 16021335 10.1007/s11103-005-7306-5

[16] Chini A, Fonseca S, Fernandez G, Adie B, Chico JM, et al (2007) The JAZ family of repressors is the missing link in jasmonate signalling. Nature 448: 666–671. 17637675 10.1038/nature06006

[17] Thines B, Katsir L, Melotto M, Niu Y, Mandaokar A, et al (2007) JAZ repressor proteins are targets of the SCF(COI1) complex during jasmonate signalling. Nature 448: 661–665. 17637677 10.1038/nature05960

[18] Fonseca S, Chini A, Hamberg M, Adie B, Porzel A, et al (2009) (+)-7-iso-Jasmonoyl-L-isoleucine is the endogenous bioactive jasmonate. Nat Chem Biol 5: 344–350. 19349968 10.1038/nchembio.161

[19] Browse J, Wager A (2012) Social network: JAZ protein interactions expand our knowledge of jasmonate signaling. Front Plant Sci 3: 1–11. 22629274 10.3389/fpls.2012.00041 PMC3355530

[20] Hou X, Lee LYC, Xia K, Yan Y, Yu H (2010) DELLAs modulate jasmonate signaling via competitive binding to JAZs. Dev Cell 19: 884–894. 21145503 10.1016/j.devcel.2010.10.024

[21] Pauwels L, Barbero GF, Geerinck J, Tilleman S, Grunewald W, et al (2010) NINJA connects the co-repressor TOPLESS to jasmonate signalling. Nature 464: 788–791. 20360743 10.1038/nature08854 PMC2849182

[22] Zhu Z, An F, Feng Y, Li P, Xue L, et al (2011) Derepression of ethylene-stabilized transcription factors (EIN3/EIL1) mediates jasmonate and ethylene signaling synergy in *Arabidopsis* . Proc Natl Acad Sci USA 108: 12539–12544. 21737749 10.1073/pnas.1103959108 PMC3145709

[23] Song S, Qi T, Huang H, Ren Q, Wu D, et al (2011) The jasmonate-ZIM domain proteins interact with the R2R3-MYB transcription factors MYB21 and MYB24 to affect jasmonate-regulated stamen development in *Arabidopsis* . Plant Cell 23: 1000–1013. 21447791 10.1105/tpc.111.083089 PMC3082250

[24] Qi T, Song S, Ren Q, Wu D, Huang H, et al (2011) The jasmonate-ZIM-domain proteins interact with the WD-Repeat/bHLH/MYB complexes to regulate jasmonate-mediated anthocyanin accumulation and trichome initiation in *Arabidopsis thaliana* . Plant Cell 23: 1795–1814. 21551388 10.1105/tpc.111.083261 PMC3123955

[25] Wild M, Davière J-M, Cheminant S, Regnault T, Baumberger N, et al (2012) The *Arabidopsis* DELLA RGA-LIKE3 is a direct target of MYC2 and modulates jasmonate signaling responses. Plant Cell 24: 3307–3319. 22892320 10.1105/tpc.112.101428 PMC3462633

[26] Shikata M, Matsuda Y, Ando K, Nishii A, Takemura M, et al (2004) Characterization of *Arabidopsis* ZIM, a member of a novel plant-specific GATA factor gene family. J Exp Bot 55: 631–639. 14966217 10.1093/jxb/erh078

[27] Vanholme B, Grunewald W, Bateman A, Kohchi T, Gheysen G (2007) The tify family previously known as ZIM. Trends Plant Sci 12: 239–244. 17499004 10.1016/j.tplants.2007.04.004

[28] Yan Y, Stolz S, Chetelat A, Reymond P, Pagni M, et al (2007) A downstream mediator in the growth repression limb of the jasmonate pathway. Plant Cell 19: 2470–2483. 17675405 10.1105/tpc.107.050708 PMC2002611

[29] Melotto M, Mecey C, Niu Y, Chung HS, Katsir L, et al (2008) A critical role of two positively charged amino acids in the Jas motif of *Arabidopsis* JAZ proteins in mediating coronatine- and jasmonoyl isoleucine-dependent interactions with the COI1 F-box protein. Plant J 55: 979–988. 18547396 10.1111/j.1365-313X.2008.03566.x PMC2653208

[30] Shyu C, Figueroa P, DePew CL, Cooke TF, Sheard LB, et al (2012) JAZ8 lacks a canonical degron and has an EAR motif that mediates transcriptional repression of jasmonate responses in *Arabidopsis* . Plant Cell 24: 536–550. 22327740 10.1105/tpc.111.093005 PMC3315231

[31] Oh Y, Baldwin IT, Galis I (2012) NaJAZh regulates a subset of defense responses against herbivores and spontaneous leaf necrosis in *Nicotiana attenuata* plants. Plant Physiol 159: 769–788. 22496510 10.1104/pp.112.193771 PMC3375940

[32] Sehr EM, Agusti J, Lehner R, Farmer EE, Schwarz M, et al (2010) Analysis of secondary growth in the *Arabidopsis* shoot reveals a positive role of jasmonate signalling in cambium formation. Plant J 63: 811–822. 20579310 10.1111/j.1365-313X.2010.04283.x PMC2988407

[33] Robson F, Okamoto H, Patrick E, Harris S-R, Wasternack C, et al (2010) Jasmonate and phytochrome a signaling in *Arabidopsis* wound and shade responses are integrated through JAZ1 stability. Plant Cell 22: 1143–1160. 20435902 10.1105/tpc.109.067728 PMC2879735

[34] Kim KC, Han J-A, Lee J, Maeng J, Hur Y (2011) Gene encoding PnFL-2 with TIFY and CCT motifs may control floral induction in *Pharbitis nil* . Genes Genom 33: 229–236.

[35] Shoji T, Ogawa T, Hashimoto T (2008) Jasmonate-induced nicotine formation in tobacco is mediated by tobacco *COI1* and *JAZ* genes. Plant Cell Physiol 49: 1003–1012. 18492687 10.1093/pcp/pcn077

[36] Sun JQ, Jiang HL, Li CY (2011) Systemin/jasmonate-mediated systemic defense signaling in tomato. Mol Plant 4: 607–615. 21357647 10.1093/mp/ssr008

[37] Demianski AJ, Chung KM, Kunkel BN (2012) Analysis of *Arabidopsis* JAZ gene expression during *Pseudomonas syringae* pathogenesis. Mol Plant Pathol 13: 46–57. 21726394 10.1111/j.1364-3703.2011.00727.x PMC6638877

[38] Ye H, Du H, Tang N, Li X, Xiong L (2009) Identification and expression profiling analysis of TIFY family genes involved in stress and phytohormone responses in rice. Plant Mol Biol 71: 291–305. 19618278 10.1007/s11103-009-9524-8

[39] Seo JS, Joo J, Kim MJ, Kim YK, Nahm BH, et al (2011) OsbHLH148, a basic helix-loop-helix protein, interacts with OsJAZ proteins in a jasmonate signaling pathway leading to drought tolerance in rice. Plant J 65: 907–921. 21332845 10.1111/j.1365-313X.2010.04477.x

[40] Ismail A, Riemann M, Nick P (2012) The jasmonate pathway mediates salt tolerance in grapevines. J Exp Bot 63: 2127–2139. 22223808 10.1093/jxb/err426 PMC3295401

[41] Zhu D, Cai H, Luo X, Bai X, Deyholos MK, et al (2012) Over-expression of a novel JAZ family gene from *Glycine soja*, increases salt and alkali stress tolerance. Biochem Biophys Res Commun 426: 273–279. 22943855 10.1016/j.bbrc.2012.08.086

[42] Shoji T, Yamada Y, Hashimoto T (2000) Jasmonate induction of putrescine N-methyltransferase genes in the root of *Nicotiana sylvestris* . Plant Cell Physiol 41: 831–839. 10965939 10.1093/pcp/pcd001

[43] Steppuhn A, Gase K, Krock B, Halitschke R, Baldwin IT (2004) Nicotine's defensive function in nature. PLoS Biol 2: e217. 15314646 10.1371/journal.pbio.0020217 PMC509292

[44] Jassbi AR, Gase K, Hettenhausen C, Schmidt A, Baldwin IT (2008) Silencing geranylgeranyl diphosphate synthase in *Nicotiana attenuata* dramatically impairs resistance to tobacco hornworm. Plant Physiol 146: 974–986. 17965175 10.1104/pp.107.108811 PMC2259063

[45] Heiling S, Schuman MC, Schoettner M, Mukerjee P, Berger B, et al (2010) Jasmonate and ppHsystemin regulate key malonylation steps in the biosynthesis of 17-hydroxygeranyllinalool diterpene glycosides, an abundant and effective direct defense against herbivores in *Nicotiana attenuata* . Plant Cell 22: 273–292. 20081114 10.1105/tpc.109.071449 PMC2828710

[46] Jongsma MA, Bakker PL, Visser B, Stiekema WJ (1994) Trypsin inhibitor activity in mature tobacco and tomato plants is mainly induced locally in response to insect attack, wounding and virus infection. Planta 195: 29–35.

[47] Jongsma MA, Bakker PL, Peters J, Bosch D, Stiekema WJ (1995) Adaptation of *Spodoptera exigua* larvae to plant proteinase inhibitors by induction of gut proteinase activity insensitive to inhibition. Proc Natl Acad Sci USA 92: 8041–8045. 7644535 10.1073/pnas.92.17.8041 PMC41282

[48] Habib H, Fazil KM (2007) Plant protease inhibitors: a defense strategy in plants. Biotechnol Mol Biol Rev 2: 63–85.

[49] Klee HJ, Clark DG (2010) Ethylene signal transduction in fruits and flowers. In: Davies PJ, editor. Plant hormones. Springer Netherlands. 377–398.

[50] Van Doorn WG (2002) Effect of ethylene on flower abscission: a survey. Ann Bot 89: 689–693. 12102524 10.1093/aob/mcf124 PMC4233834

[51] Dubos C, Stracke R, Grotewold E, Weisshaar B, Martin C, et al (2010) MYB transcription factors in *Arabidopsis* . Trends Plant Sci 15: 573–581. 20674465 10.1016/j.tplants.2010.06.005

[52] Colquhoun TA, Schwieterman ML, Wedde AE, Schimmel BC, Marciniak DM, et al (2011) EOBII controls flower opening by functioning as a general transcriptomic switch. Plant Physiol 156: 974–984. 21464473 10.1104/pp.111.176248 PMC3177291

[53] Liu G, Thornburg RW (2012) Knockdown of MYB305 disrupts nectary starch metabolism and floral nectar production. Plant J 70: 377–388. 22151247 10.1111/j.1365-313X.2011.04875.x

[54] Sablowski RWM, Moyano E, Culianez-Macia FA, Schuch W, Martin C, et al (1994) A flower-specific Myb protein activates transcription of phenylpropanoid biosynthetic genes. EMBO J 13: 128–137. 8306956 10.1002/j.1460-2075.1994.tb06242.x PMC394786

[55] Liu G, Ren G, Guirgis A, Thornburg RW (2009) The MYB305 transcription factor regulates expression of nectarin genes in the ornamental tobacco floral nectary. Plant Cell 21: 2672–2687. 19783761 10.1105/tpc.108.060079 PMC2768911

[56] Bunker TW, Koetje DS, Stephenson LC, Creelman RA, Mullet JE, et al (1995) Sink limitation induces the expression of multiple soybean vegetative lipoxygenase mRNAs while the endogenous jasmonic acid level remains low. Plant Cell 7: 1319–1331. 7549487 10.1105/tpc.7.8.1319 PMC160954

[57] Creelman RA, Mullet JE (1997) Biosynthesis and action of jasmonates in plants. Annu Rev Plant Physiol Plant Mol Biol 48: 355–381. 15012267 10.1146/annurev.arplant.48.1.355

[58] Chung HS, Howe GA (2009) A critical role for the TIFY motif in repression of jasmonate signaling by a stabilized splice variant of the JASMONATE ZIM-domain protein JAZ10 in *Arabidopsis* . Plant Cell 21: 131–145. 19151223 10.1105/tpc.108.064097 PMC2648087

[59] Clark DG, Richards C, Hilioti Z, Lind-Iversen S, Brown K (1997) Effect of pollination on accumulation of ACC synthase and ACC oxidase transcripts, ethylene production and flower petal abscission in geranium (*Pelargonium* × *hortorum* L.H. Bailey). Plant Mol Biol 34: 855–865. 9290638 10.1023/a:1005877809905

[60] Wilkinson JQ, Lanahan MB, Clark DG, Bleecker AB, Chang C, et al (1997) A dominant mutant receptor from *Arabidopsis* confers ethylene insensitivity in heterologous plants. Nat Biotechnol 15: 444–447. 9131623 10.1038/nbt0597-444

[61] Jones ML (2008) Ethylene signaling is required for pollination-accelerated corolla senescence in petunias. Plant Sci 175: 190–196.

[62] Bhattacharya S, Baldwin IT (2012) The post-pollination ethylene burst and the continuation of floral advertisement are harbingers of non-random mate selection in *Nicotiana attenuata* . Plant J 71: 587–601. 22458597 10.1111/j.1365-313X.2012.05011.x

[63] Shimizu-Yumoto H, Ichimura K (2012) Effects of ethylene, pollination, and ethylene inhibitor treatments on flower senescence of gentians. Postharvest Biol Technol 63: 111–115.

[64] McConn M, Browse J (1996) The critical requirement for linolenic acid is pollen development, not photosynthesis, in an *Arabidopsis* mutant. Plant Cell 8: 403–416. 12239389 10.1105/tpc.8.3.403 PMC161109

[65] Mandaokar A, Thines B, Shin B, Markus Lange B, Choi G, et al (2006) Transcriptional regulators of stamen development in *Arabidopsis* identified by transcriptional profiling. Plant J 46: 984–1008. 16805732 10.1111/j.1365-313X.2006.02756.x

[66] Xie D (1998) COI1: an *Arabidopsis* gene required for jasmonate-regulated defense and fertility. Science 280: 1091–1094. 9582125 10.1126/science.280.5366.1091

[67] Paschold A, Bonaventure G, Kant MR, Baldwin IT (2008) Jasmonate perception regulates jasmonate biosynthesis and JA-Ile metabolism: the case of COI1 in *Nicotiana attenuata* . Plant Cell Physiol 49: 1165–1175. 18559356 10.1093/pcp/pcn091

[68] Carter C, Thornburg RW (2004) Is the nectar redox cycle a floral defense against microbial attack? Trends Plant Sci 9: 320–324. 15231276 10.1016/j.tplants.2004.05.008

[69] Horner HT, Healy RA, Ren G, Fritz D, Klyne A, et al (2007) Amyloplast to chromoplast conversion in developing ornamental tobacco floral nectaries provides sugar for nectar and antioxidants for protection. Am J Bot 94: 12–24. 21642203 10.3732/ajb.94.1.12

[70] Krügel T, Lim M, Gase K, Halitschke R, Baldwin IT (2002) *Agrobacterium*-mediated transformation of *Nicotiana attenuata*, a model ecological expression system. Chemoecology 12: 177–183.

[71] Bubner B, Gase K, Berger B, Link D, Baldwin IT (2006) Occurrence of tetraploidy in *Nicotiana attenuata* plants after *Agrobacterium*-mediated transformation is genotype specific but independent of polysomaty of explant tissue. Plant Cell Rep 25: 668–675. 16518637 10.1007/s00299-005-0111-4

[72] Von Dahl CC, Winz RA, Halitschke R, Kühnemann F, Gase K, et al (2007) Tuning the herbivore-induced ethylene burst: the role of transcript accumulation and ethylene perception in *Nicotiana attenuata* . Plant J 51: 293–307. 17559506 10.1111/j.1365-313X.2007.03142.x

[73] Kistner C, Matamoros M (2005) RNA isolation using phase extraction and LiCl precipitation. *Lotus japonicus* Handbook: 123–124.

[74] Kallenbach M, Gilardoni PA, Allmann S, Baldwin IT, Bonaventure G (2011) C12 derivatives of the hydroperoxide lyase pathway are produced by product recycling through lipoxygenase-2 in *Nicotiana attenuata* leaves. New Phytol 191: 1054–1068. 21615741 10.1111/j.1469-8137.2011.03767.x

[75] Tusher VG, Tibshirani R, Chu G (2001) Significance analysis of microarrays applied to the ionizing radiation response. Proc Natl Acad Sci USA 98: 5116–5121. 11309499 10.1073/pnas.091062498 PMC33173

