# Supplementary figures and images for: A Jasmonate ZIM-Domain Protein NaJAZd Regulates Floral Jasmonic Acid Levels and Counteracts Flower Abscission in Nicotiana attenuata Plants
Source: PLoS One. 2013 Feb 28;8(2):e57868. doi: 10.1371/journal.pone.0057868 (PMC3585257; doi:10.1371/journal.pone.0057868)

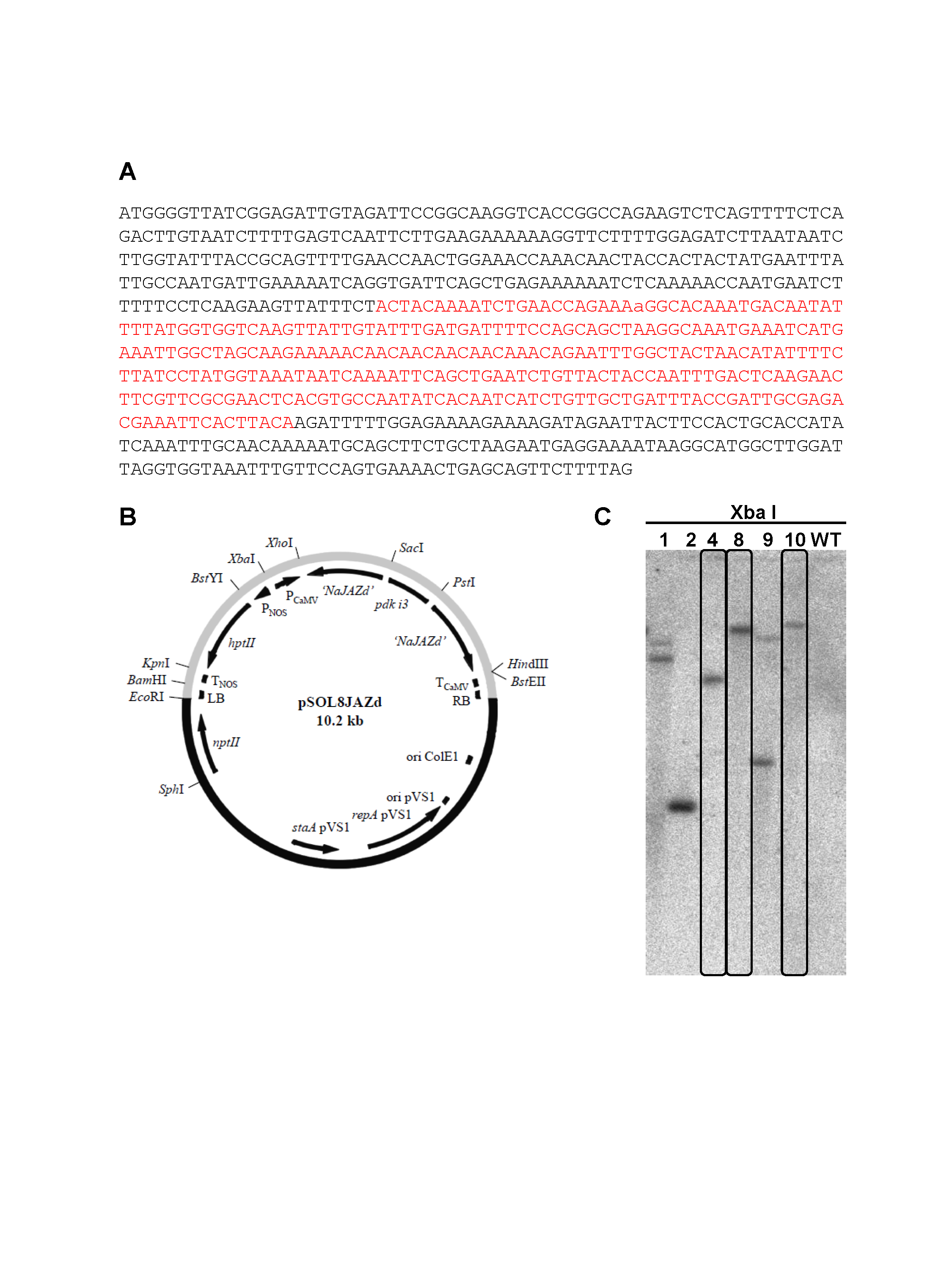

Supplement: Figure S1 — Generation of stable NaJAZd -silenced N. attenuata plants. (A) A 303 bp region in NaJAZd gene used for gene silencing is shown in red letters. (B) The pSOL8JAZd vector containing inverted repeat of NaJAZd gene used for Agrobacterium tumefaciens-mediated transformation and generation of stably silenced N. attenuata irJAZd plants. (C) Southern blot analysis of 6 independently transformed irJAZd (irJAZd-1, -2, -4, -8, -9, and -10) lines and WT. The genomic DNA was digested with XbaI enzyme and hybridized with a 32P-labeled probe coding for the hygromycin resistance gene located between the right and left T-DNA borders of the transformation vector pSOL8JAZd. The black boxes indicate single T-DNA insertion lines selected for further experiments: irJAZd-4, -8, and -10. (TIF) [file pone.0057868.s001.tif]

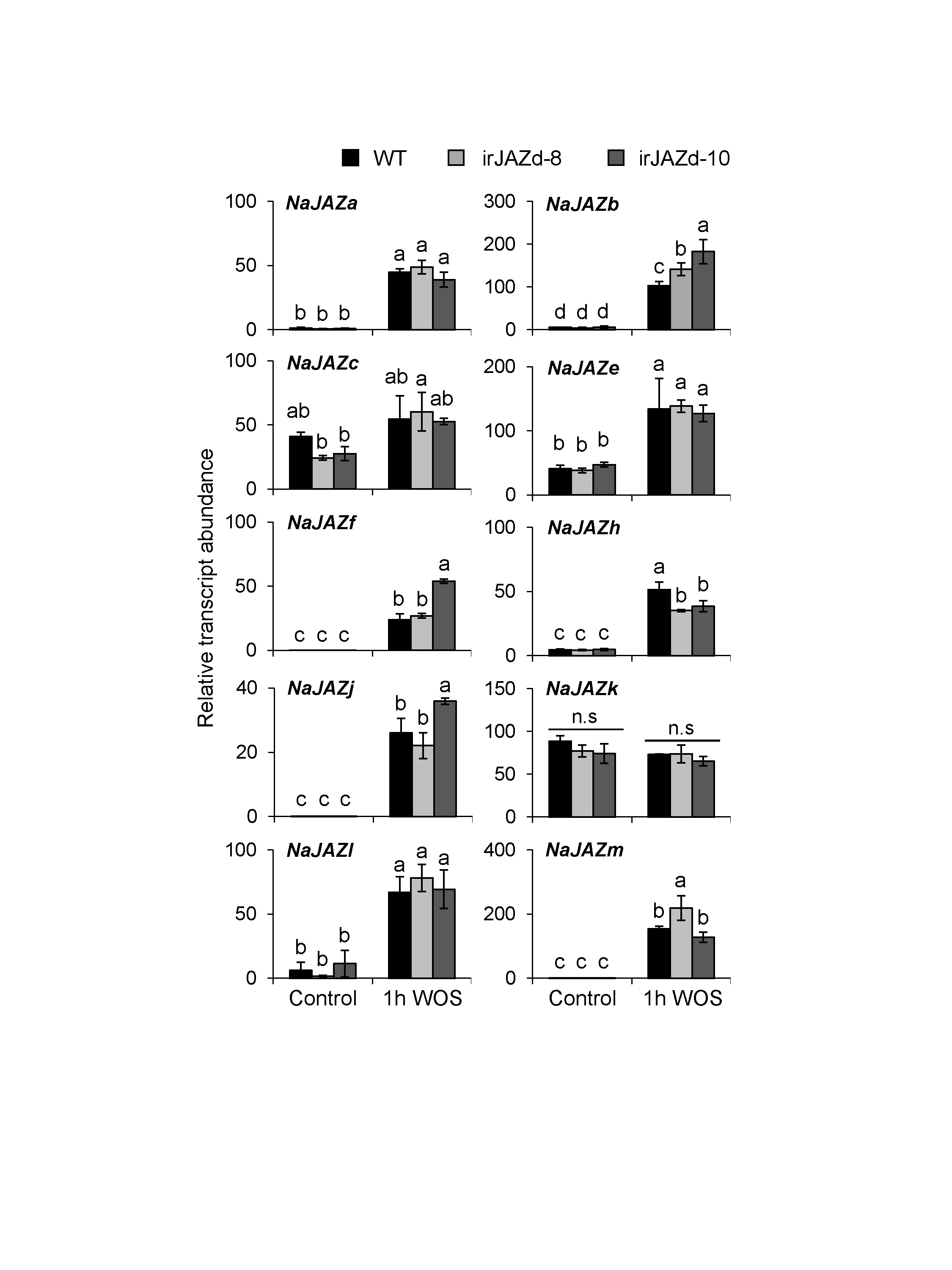

Supplement: Figure S2 — Transcript abundances of other NaJAZ genes in irJAZd plants. Transcript abundances of other NaJAZ genes were determined by qPCR in the leaves of irJAZd and WT plants before and 1 h after W+OS elicitation; bars indicate EF1α-normalized relative transcript abundances ± SE (n = 3) and different letters indicate significant differences among the combination of genotypes (WT vs. independent NaJAZd-silenced lines, irJAZd-8, 10) and treatments determined by one-way-ANOVA (P≤0.05); n.s, not significantly different. (TIF) [file pone.0057868.s002.tif]

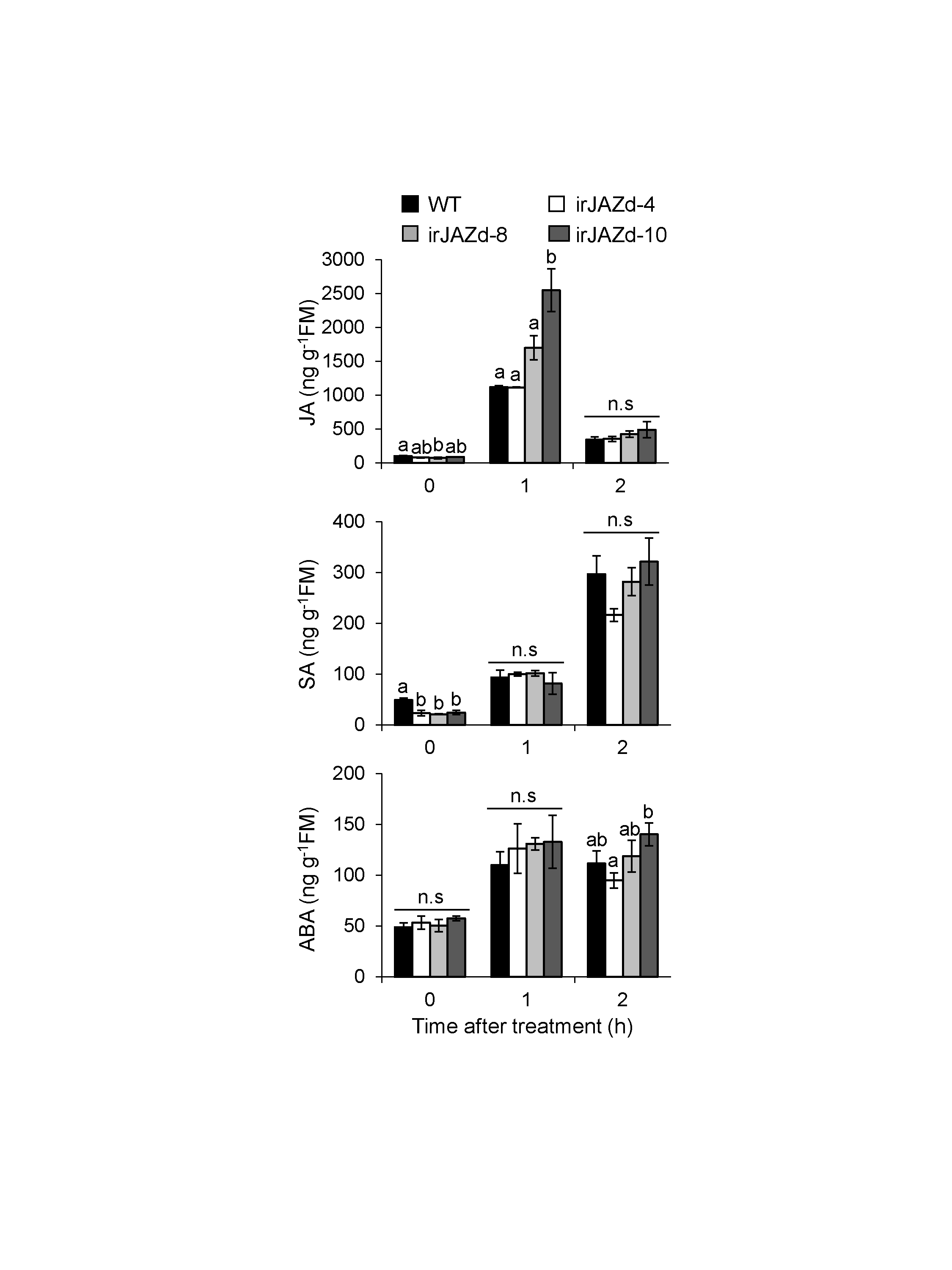

Supplement: Figure S3 — NaJAZd -silencing does not significantly alter basal or herbivory-induced phytohormones levels. Rosette stage plants of WT and irJAZd (irJAZd-4, -8 and -10) were treated with W+OS and harvested before, 1 and 2 h after treatment. Mean ± SE levels of JA, ABA and SA (n = 3) were determined by LC- ESI-MS/MS using internal deuterium-labeled phytohormone standards. Different letters indicate significant differences among the different genotypes (WT vs. independent NaJAZd silenced lines, irJAZd-4, -8, 10) at the same time points by ANOVA (P≤0.05); n.s, not significantly different. FM, fresh mass. (TIF) [file pone.0057868.s003.tif]

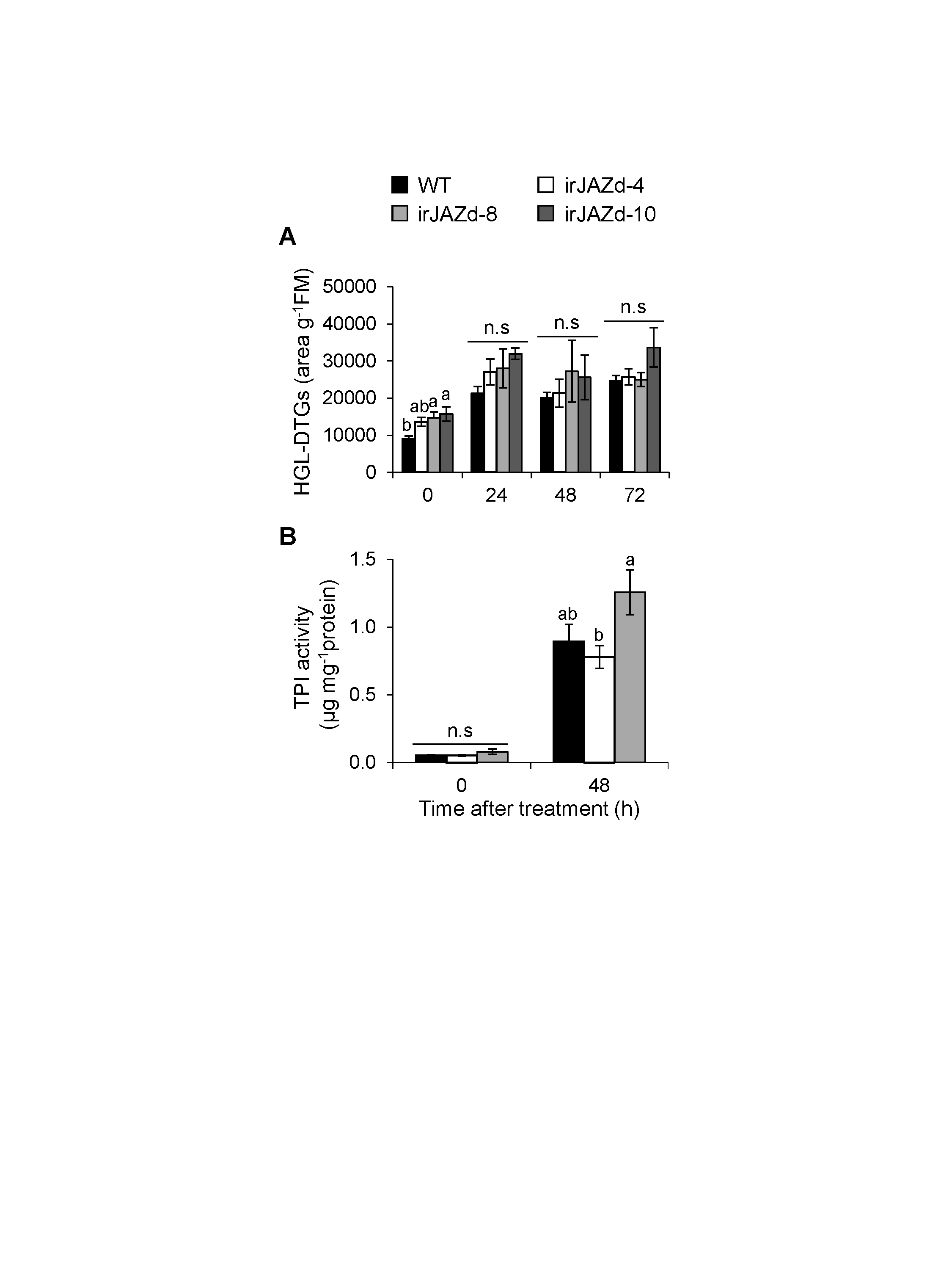

Supplement: Figure S4 — NaJAZd -deficiency does not affect levels of defense-related secondary metabolites, HGL-DTGs and TPIs, in irJAZd plants. Rosette stage WT and irJAZd (irJAZh-4, -8 and -10) plants were treated with W+OS and harvested before and 24, 48, and 72 h after treatment for determination of total HGL-DTGs levels and trypsin protease inhibitors (TPIs) activity. (A) Mean ± SE levels of total HGL-DTGs measured by HPLC coupled to ELS (Evaporative Light Scattering) detector (n = 3). (B) Mean ± SE levels of TPI activities determined by radial diffusion assay (n = 3). Different letters in A and B indicate significant differences among the different genotypes (WT vs. independent NaJAZd silenced lines, irJAZd-4, -8, 10) at the same time point determined by one-way-ANOVA (P≤0.05); n.s, not significantly different. FM, fresh mass. (TIF) [file pone.0057868.s004.tif]

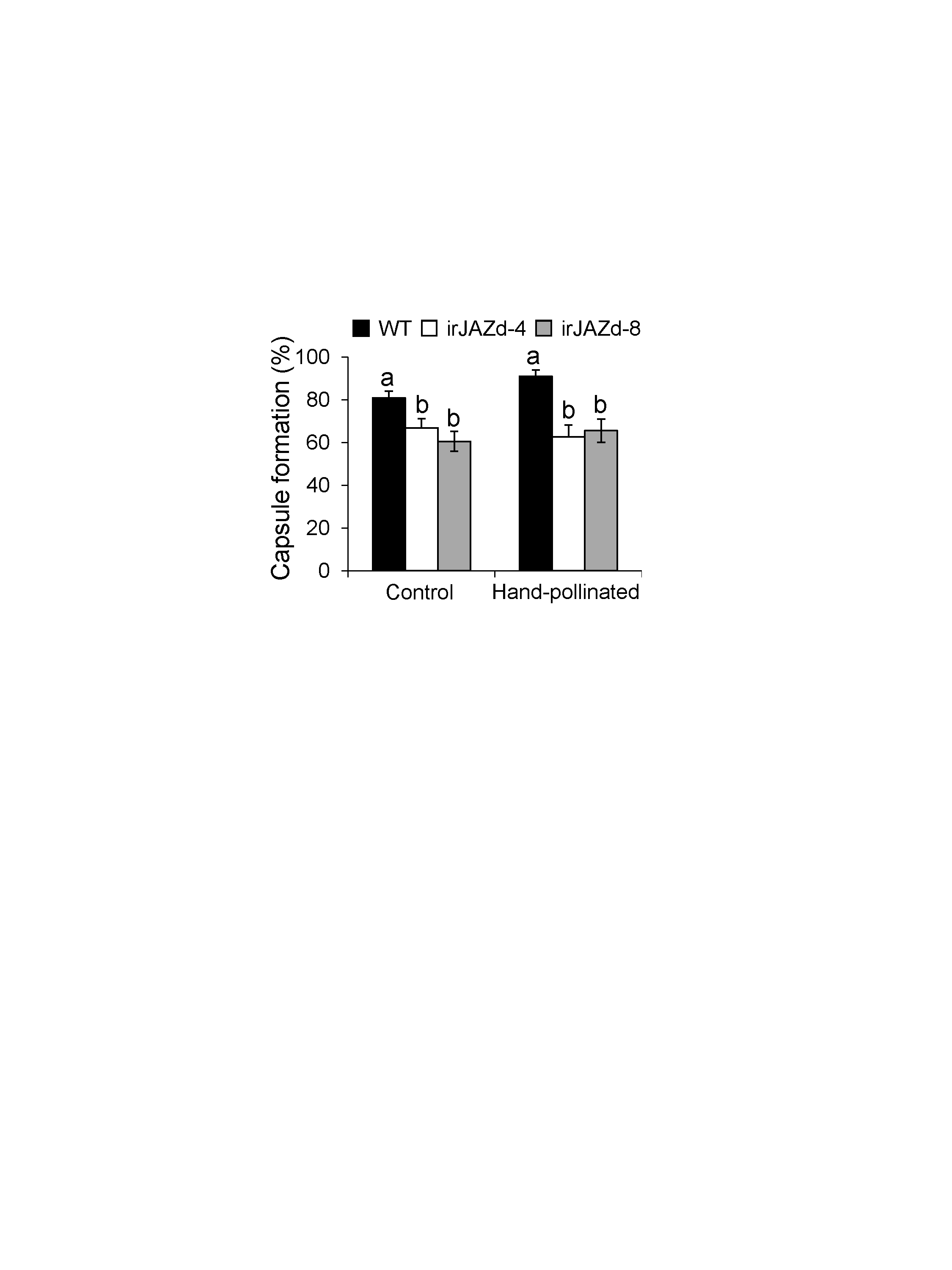

Supplement: Figure S5 — Hand-pollination does not rescue seed capsule formation in irJAZd plants. Plants were kept in the glasshouse until flowering stage (approximately 55 d after germination) and, in the previous evening, all fully elongated flowers ready to open next morning were labeled with color strings. In half of the plants, hand- pollination was conducted while second half remained exclusively self-pollinated. Approximately 10 d later, mature seed capsules resulting from labeled flowers in each group were counted and percentage of capsules originating from self- and hand-pollination groups of WT and irJAZd plants were determined (n = 24). Different letters indicate significant differences among the different genotypes (WT vs. independent NaJAZd-silenced lines, irJAZd-4, -8) at the same condition determined by one-way-ANOVA (P≤0.05). (TIF) [file pone.0057868.s005.tif]

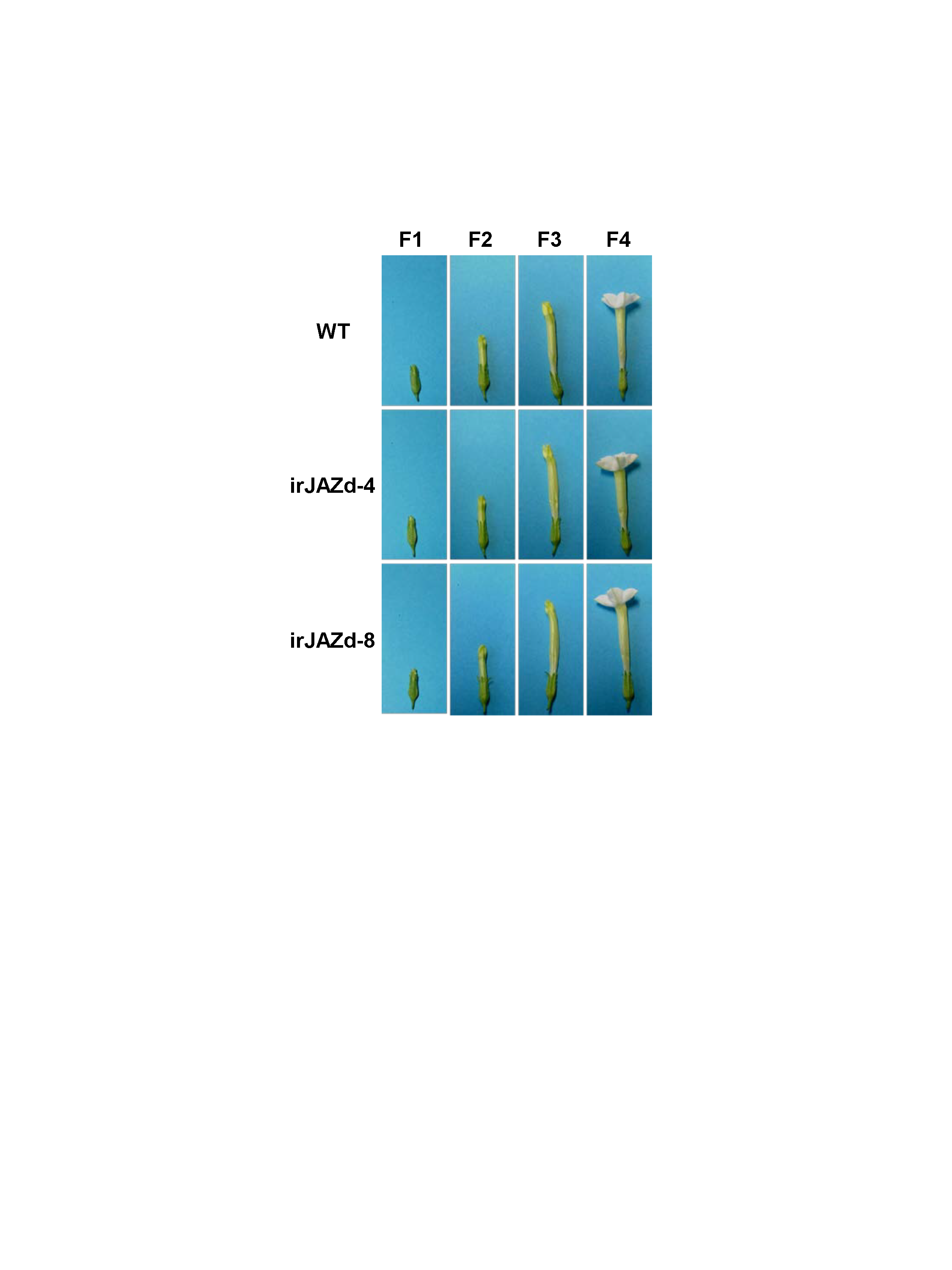

Supplement: Figure S6 — Flowers and buds of irJAZd transgenic and WT plants at F1–F4 stages of development. Flower buds and flowers were detached from 57-d-old plants and photographed to capture the highly similar morphology of flowers in two transgenic irJAZd-4 and -8 lines and WT. (TIF) [file pone.0057868.s006.tif]

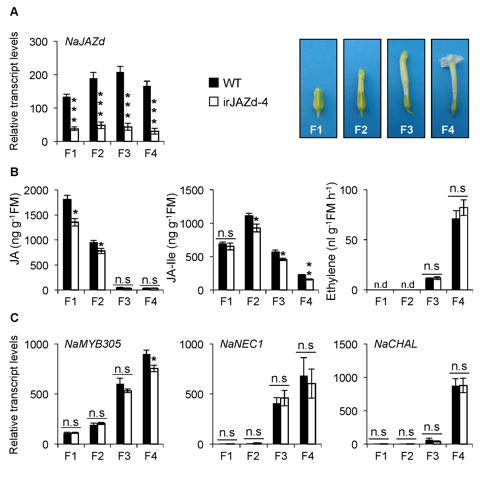

Supplement: Figure S7 — NaJAZd regulates phytohormone levels and flower development-related genes. WT and irJAZd-4 plants were grown in glasshouse and four different developmental stages of flowers (F1, F2, F3, and F4) were collected 57 d after germination. (A) Transcript abundances of NaJAZd determined by qPCR in irJAZd-4 flowers were significantly lower compared to WT. (B) Mean JA and JA-Ile levels ± SE determined by LC-ESI-MS/MS using four individual stage flowers and mean ethylene levels ± SE measured by photoacoustic spectrometer using a mixture of five flowers of each stage. (C) Transcripts abundances of flower development-related genes, NaMYB305, NaNEC1, and NaCHAL determined by qPCR: irJAZd-4 plants were impaired in expression of NaMYB305 gene in last stage of flower development (F4) while NaNEC1 and NaCHAL transcripts were unaltered in irJAZd-4 compared to WT flowers. Bars ± SE in (C) show EF1α-normalized relative transcript abundances. Statistical differences in phytohormones, JA, JA-Ile, ethylene (n = 4), and transcript abundances (n = 4) were determined by Student t-test. Asterisks represent significant differences between WT and irJAZd in same stage of flowers (*P≤0.05, **P≤0.01, ***P≤0.001); n.s, not significantly different; FM, fresh mass. (TIFF) [file pone.0057868.s007.tif]
