# Supplementary material for: A Jasmonate ZIM-Domain Protein NaJAZd Regulates Floral Jasmonic Acid Levels and Counteracts Flower Abscission in Nicotiana attenuata Plants
Source: PLoS One. 2013 Feb 28;8(2):e57868. doi: 10.1371/journal.pone.0057868 (PMC3585257; doi:10.1371/journal.pone.0057868)
Supplement: Table S1 — Up-regulated genes in irJAZd plants compared to WT plants determined by microarrays. (PDF) [file pone.0057868.s008.pdf]

Table S1. Up-regulated genes in irJAZd plants compared to WT plants determined by microarrays

Classification	Fold Change	Bincode (TAIR)	Annotation	Gene ID	ACC number
**TCA / org. transformation.**	4.30	8.3	Carbonate dehydratase	Na_454_18630	P27141
**Miscellaneous enzyme families**	4.39	26.12	Peroxidase	Na_454_32590	Q9XIV8
**RNA.regulation of transcription**	4.11	27.3.5	ARR17 (ARABIDOPSIS RESPONSE REGULATOR 17)	Na_454_26424	NP_567037
	3.49	27.3.99	Leucine zipper factor-related	Na_454_15857	NP_563798
**DNA.synthesis/chromatin structure**	3.72	28.1.3	Histone H1	Na_454_08010	AAC41651
**Protein.targeting.nucleus**	3.02	29.3.1	IMPORTIN ALPHA ISOFORM 2	Na_454_15992	ABM05488
**Protein.postranslational modification**	3.05	29.4	Protein kinase	Na_454_34744	CAA50374
**Signalling.receptor kinases**	3.50	30.2.26	CCR1 (ARABIDOPSIS THALIANA CRINKLY4 RELATED 1)	Na_454_31246	NP_187589

Table S1. (continued)

Classification	Fold Change	Bincode (TAIR)	Annotation	Gene ID	ACC number
Not assigned	26.28	35.1	Carbon-nitrogen hydrolase family protein	Na_454_20897	NP_196765
	4.85	35.1	Carbon-nitrogen hydrolase family protein	Na_454_17587	NP_196765
	3.00	35.1	Glycosyl transferase family 1 protein	Na_454_37011	NP_173105

* Microarray data is processed by SAM after 75% percentile normalization (FDR=2.09).
* Up-regulated genes were determined by a greater than 3-fold induction of normalized signals in their expression ratio (irJAZd / WT) of 2h-WOS treated leaves. The values are the average ratio of 3 biological replicates of the microarrays.
* All changes in gene expression were statistically significant by t-test ($P < 0.05$)
* Gene annotation is processed by Blast X (E-value < 1e-5)
* Classification of genes is based on GO classification from TAIR (http://www.arabidopsis.org).
