# Supplementary material for: A Jasmonate ZIM-Domain Protein NaJAZd Regulates Floral Jasmonic Acid Levels and Counteracts Flower Abscission in Nicotiana attenuata Plants
Source: PLoS One. 2013 Feb 28;8(2):e57868. doi: 10.1371/journal.pone.0057868 (PMC3585257; doi:10.1371/journal.pone.0057868)
Supplement: Table S2 — Down-regulated genes in irJAZd plants compared to WT plants determined by microarrays. (PDF) [file pone.0057868.s009.pdf]

Table S2. Down-regulated genes in irJAZd plants compared to WT plants determined by microarrays

Classification	Fold Change	Bincode (TAIR)	Annotation	Gene ID	ACC number
**Major CHO metabolism**	3.86	2.2.1.1	Fructokinase	Na_454_06965	NP_001234206
**Minor CHO metabolism**	3.76	3.60	Putative beta-1,3-glucan synthase	Na_454_25961	AAK49452
**Glycolysis**	3.70	4.2.5	6-phosphofructokinase 4	Na_454_38726	NP_001032120
**Cell wall**	3.79	10.1.9	UDP-glucose 4-epimerase	Na_454_18676	AAP42567
	3.28	10.5.2	Proline-rich protein	Na_454_40169	AAF28387
**Amino acid metabolism**	5.02	13.1.3.1.1	Asparagine synthetase 3	Na_454_21256	NP_001031864
	3.19	13.2.3.4	S-adenosyl-L-homocysteine hydrolase	Na_454_21170	AAV31754
**Secondary metabolism**	4.17	16.8.3	Glycosyltransferase	Na_454_07441	ADI33725
	2.99	16.4.1	Iron ion binding / oxidoreductase/ oxidoreductase protein	Na_454_22715	NP_190233
**Hormone metabolism**	3.07	17.7.1.2	Lipoxygenase (NaLOX1b)	Na_454_37768	AAP83135
**Stress.abiotic**	3.67	20.2.1	DnaJ-like protein isoform	Na_454_21360	ABI34703

Table S2. (continued)

Classification	Fold Change	Bincode (TAIR)	Annotation	Gene ID	ACC number
**Biodegradation of Xenobiotics**	3.37	24.10	Glyoxalase II 3	Na_454_20653	NP_564636
**Miscellaneous enzyme families**	3.18	26.10	Cytochrome P450, family 78, subfamily A, polypeptide 10	Na_454_31815	NP_177551
**RNA.regulation of transcription**	8.13	27.3.22	Class 2 knotted1-like protein	Na_454_34641	BAF95776
**RNA.RNA binding**	3.64	27.40	RNA recognition motif-containing protein	Na_454_41880	NP_973888
**Protein.synthesis**	3.31	29.2.3	Eukaryotic translation initiation factor SUI1-like protein	Na_454_11378	NP_177291
**Protein.postranslational modification**	3.55	29.40	Protein kinase family protein-like	Na_454_17022	BAD61275
	3.48	29.40	O-methyltransferase	Na_454_25230	AAA34088
	9.47	29.4.1	Putative serine/threonine protein kinase	Na_454_15886	AAC69450
	3.28	29.4.1	MAP kinase, putative	Na_454_21759	AAM20643
**Protein.degradation**	3.39	29.5.11.4.2	RING/U-box domain-containing protein	Na_454_30344	NP_175132
	3.20	29.5.11.4.2	E3 ubiquitin-protein ligase ATL41	Na_454_21141	NP_181765
	3.71	29.5.5	Serine carboxypeptidase-like 16	Na_454_04811	NP_566414
**Signalling**	3.95	30.1.1	PAR-1c (photoassimilate-responsive 1c)	Na_454_25707	CAA58732
	3.30	30.11	Phytochrome F	Na_454_38145	AAC49301
	2.96	30.2.99	Leucine-rich repeat-containing protein	Na_454_03038	NP_188563
	4.25	30.30	Calcium-binding EF-hand domain-containing protein	Na_454_06154	NP_564623

Table S2. (continued)

Classification	Fold Change	Bincode (TAIR)	Annotation	Gene ID	ACC number
**Development**	5.36	33.99	Nodulin MtN3 family protein	Na_454_41228	NP_200131
	3.44	33.99	NAC domain protein NAC2	Na_454_11638	ABK96797
	3.06	33.30	Squamosa promoter binding-like protein	Na_454_11778	ABH07904
**Transporter.sugars**	4.93	34.20	Bidirectional sugar transporter SWEET3	Na_454_16634	XP_002267886
**Not assigned**	11.02	35.20	Protein LURP-one-related 5	Na_454_11287	NP_178129
	9.35	35.20	Jasmonate ZIM-domain protein 1	Na_454_02978	BAG68655
	6.10	35.20	Putative non-LTR retrolelement reverse transcriptase	Na_454_31619	AAD26953
	4.65	35.20	UDP-glycosyltransferase family protein	Na_454_43115	NP_001190226
	3.09	35.20	Ninja-family protein AFP2	Na_454_29306	NP_563933
	3.72	35.10	Rho GTPase activation protein (RhoGAP) with PH domain	Na_454_36722	NP_196776
	3.64	35.1.5	Pentatricopeptide repeat-containing protein	Na_454_25757	XP_002273247

* Microarray data is processed by SAM after 75% percentile normalization (FDR=2.09).
* Down-regulated genes were determined by a greater than 3-fold repression of normalized signals in their expression ratio (irJAZd / WT) of 2h-WOS treated leaves. The values are the average ratio of 3 biological replicates of the microarrays.
* All changes in gene expression were statistically significant by t-test ($P < 0.05$)
* Gene annotation is processed by Blast X (E-value < 1e-5)
* Classification of genes is based on GO classification from TAIR (http://www.arabidopsis.org).
