# Supplementary material for: A Jasmonate ZIM-Domain Protein NaJAZd Regulates Floral Jasmonic Acid Levels and Counteracts Flower Abscission in Nicotiana attenuata Plants
Source: PLoS One. 2013 Feb 28;8(2):e57868. doi: 10.1371/journal.pone.0057868 (PMC3585257; doi:10.1371/journal.pone.0057868)
Supplement: Table S3 — Primer sequences used in quantitative real time PCR (qPCR). (PDF) [file pone.0057868.s010.pdf]

Table S3. Primer sequences used in quantitative real time PCR (qPCR)

Gene name	Primer sequence
NaJAZa FP	CAGTGAAAGCTGAGCAATTCTAGTACTC
NaJAZa RP	AGCCTTAGACGAATTGAATACCTACAC
NaJAZb FP	GGGAAAGCTAATTCAAGACAAATGG
NaJAZb RP	TGTATTCTTTAGCCACCAATCTAGC
NaJAZc FP	GGAAAGGGTGATGAACGCTGCA
NaJAZc RP	TATGGCAATAGGCTGCCTTCAGAC
NaJAZd FP	GAGATTGTAGATTCCGGCAAGGTCA
NaJAZd RP	TTCTCAGCTGAATCACCTGA
NaJAZe FP	CAATTTGGTCAAGGAGACGTGA
NaJAZe RP	GGCTCTGATCACAATTACAAGG
NaJAZf FP	CAAGTAGAGAAATGGAGGAG
NaJAZf RP	GCTAGTGATGATATGGAGAAG
NaJAZh FP	TTCTGCTACGCCGCAAGTACTG
NaJAZh RP	GGTATGGCGCTCTAGCCGTTG
NaJAZj FP	CATCATCACCAATTTCAGAGCCTTC
NaJAZj RP	TCCAATTTTCCAATTTCTCCCAGCA
NaJAZk FP	TCTATGGTGATGTGCCTGCTGAC
NaJAZk RP	AACGGATATCCAAGCTAGCTGTTG
NaJAZl FP	TTGCCAGAAGGAAATCCCTGAAGAG
NaJAZl RP	TCCATCAAAAGCTAGCCCTACTTAGC
NaJAZm FP	AGTGCGTCAAATTTGAGAGCACCA
NaJAZm RP	GCTGCTTGAATCCTCCTTTCTCTTC
NaMYB305	FP ATGCTAAGTGGGGAAACAG
NaMYB305	RP GCAATTGCATGGACCAGA
NaNEC1 FP	TGCTGTTTTTGCCGCTCCTT
NaNEC1 RP	ACCACATCGTGGCACAGAGAGT
NaCHAL FP	TTCACGTTTCAAGGCCCAA
NaCHALRP	TGCTCCATCAGCGAAAAGG
NtEF1a FP	CCACACTTCCCACATTGCTGTCA
NtEF1a RP	CGCATGTCCCTCACAGCAAAAC
