# Supplementary material for: A Jasmonate ZIM-Domain Protein NaJAZd Regulates Floral Jasmonic Acid Levels and Counteracts Flower Abscission in Nicotiana attenuata Plants
Source: PLoS One. 2013 Feb 28;8(2):e57868. doi: 10.1371/journal.pone.0057868 (PMC3585257; doi:10.1371/journal.pone.0057868)
Supplement: Table S4 — QC table showing technical variability of 30 selected control genes spotted in 10 different locations on microarray chips. (PDF) [file pone.0057868.s011.pdf]

Table S4. QC table showing technical variability of 30 selected control genes spotted in 10 different locations on microarray chips.

The expression values were normalized by 75 percentile and log 2 transformed before calculation average and SE of technical replicates.

Gene ID	WT-1	WT-2	WT-3	WT-4	WT-5	WT-6
**Na_454_00153**	3.102678	5.228659	5.308914	5.49396	5.510409	5.452788
Na_454_00153	5.430365	5.292221	5.357254	5.490642	5.550115	5.552954
Na_454_00153	5.269469	5.25465	5.301848	5.484963	5.446494	5.490409
Na_454_00153	4.916878	5.189664	5.322145	5.417511	5.428358	5.389279
Na_454_00153	5.428364	5.318018	5.472222	5.606294	5.58202	5.659425
Na_454_00153	5.463539	5.309923	5.455644	5.542436	5.565891	5.533057
Na_454_00153	5.390505	5.354294	5.448306	5.488975	5.547724	5.567554
Na_454_00153	5.476523	5.325844	5.480719	5.513835	5.584381	5.618717
Na_454_00153	5.33067	5.331533	5.408558	5.486622	5.492589	5.523511
Na_454_00153	5.411647	5.191409	5.295528	5.453557	5.456115	5.436405
**Average**			5.258126			5.519293
**Standard Error (SE)**			0.07736			0.011677
**Na_454_00163**	3.053905	2.30161	2.172045	2.866999	2.305365	2.440011
Na_454_00163	3.110707	2.281886	2.075472	2.846393	2.287889	2.382794
Na_454_00163	2.823222	2.258472	2.138299	2.895422	2.416752	2.474895
Na_454_00163	3.008461	2.334496	2.163386	2.952028	2.430628	2.52776
Na_454_00163	3.129168	2.314589	2.116558	2.841177	2.332064	2.497375
Na_454_00163	3.155948	2.31619	2.10716	2.932141	2.394498	2.477966
Na_454_00163	2.099214	2.385068	2.176239	2.923705	2.332651	2.525688
Na_454_00163	3.113678	2.397269	2.170223	2.845356	2.363957	2.490758
Na_454_00163	3.047987	2.298073	2.079237	2.882196	2.307956	2.530867
Na_454_00163	3.014164	2.337978	2.123562	2.846731	2.349226	2.442611
**Average**			2.467724			2.57427
**Standard Error (SE)**			0.073039			0.042813
**Na_454_00262**	3.899846	3.73093	3.815086	3.793945	3.551883	3.652264
Na_454_00262	3.992699	3.679946	3.871049	3.783207	3.60817	3.687914
Na_454_00262	4.034709	3.878909	3.879799	3.856493	3.74462	3.826788
Na_454_00262	3.904221	3.868207	3.956163	3.94011	3.726371	3.880254
Na_454_00262	3.986818	3.83919	3.94133	3.888351	3.799067	3.848197
Na_454_00262	4.120827	3.824172	3.930624	3.875133	3.756608	3.743249
Na_454_00262	4.028203	3.79984	3.897945	3.906127	3.730101	3.937469
Na_454_00262	3.910087	3.69151	3.84856	3.821731	3.487665	3.788116
Na_454_00262	3.594781	3.736451	3.860198	3.836235	3.68536	3.800487
Na_454_00262	3.306461	3.75324	3.872212	3.806136	3.661463	3.794204
**Average**			3.871189			3.776145
**Standard Error (SE)**			0.027841			0.019738
**Na_454_00266**	3.685713	3.719355	3.792743	3.685153	3.748467	3.529984
Na_454_00266	3.567359	3.596745	3.649512	3.548218	3.655543	3.535064
Na_454_00266	3.661725	3.642833	3.694071	3.565923	3.680516	3.520905
Na_454_00266	3.614821	3.60198	3.641692	3.568416	3.616361	3.555893
Na_454_00266	3.68889	3.605609	3.716483	3.526588	3.694692	3.568823
Na_454_00266	3.666982	3.72079	3.736941	3.588186	3.673631	3.629661
Na_454_00266	3.768677	3.597094	3.668749	3.676811	3.729046	3.624144

Na_454_00266	3.693249	3.64711	3.701041	3.651739	3.65495	3.60132
Na_454_00266	3.000039	3.712908	3.792671	3.787051	3.790793	3.688733
Na_454_00266	3.509887	3.535915	3.605654	3.551726	3.616813	3.561837
**Average**			3.651325			3.633208
**Standard Error (SE)**			0.025448			0.014078
**Gene ID**	**WT-1**	**WT-2**	**WT-3**	**WT-4**	**WT-5**	**WT-6**
**Na_454_00283**	6.15586	5.987585	6.03658	6.038282	5.753025	5.909941
Na_454_00283	6.159869	6.062705	6.183249	6.134803	5.948862	6.035026
Na_454_00283	6.141579	6.048924	6.152547	6.060539	5.916054	6.074827
Na_454_00283	6.297681	5.983136	6.091855	6.058118	5.957795	6.037959
Na_454_00283	6.332432	5.943332	6.053139	6.023735	5.946969	5.975434
Na_454_00283	6.473475	5.968147	6.091743	6.052683	6.031797	5.951704
Na_454_00283	6.305056	5.990674	6.129752	6.029588	5.941688	6.001703
Na_454_00283	6.059835	5.776007	5.851518	5.893396	5.809078	5.827014
Na_454_00283	5.666056	5.957224	6.106958	6.061157	5.883002	5.970551
Na_454_00283	6.051437	5.821326	5.909281	5.852762	5.79571	5.755533
**Average**			6.07433			5.97499
**Standard Error (SE)**			0.030864			0.018421
**Na_454_00339**	3.174038	3.182305	3.133584	2.978966	3.67459	3.00232
Na_454_00339	3.080644	3.0013	3.023766	2.924529	3.613722	2.912544
Na_454_00339	3.148295	3.084623	3.087538	2.996975	3.642317	2.882724
Na_454_00339	3.129536	3.113567	3.11513	2.945871	3.73888	2.893432
Na_454_00339	3.120714	3.108026	3.153317	2.965508	3.746549	2.955777
Na_454_00339	3.108352	3.039104	3.107209	2.962883	3.729468	2.925756
Na_454_00339	3.040867	3.094054	3.218249	3.032304	3.755566	3.043778
Na_454_00339	3.230226	3.109968	3.160601	3.022088	3.739735	2.93085
Na_454_00339	3.101818	3.084814	3.073078	3.018594	3.679108	3.046344
Na_454_00339	3.02894	3.004016	3.029776	2.951918	3.657354	2.912456
**Average**			3.112027			3.213377
**Standard Error (SE)**			0.010647			0.064753
**Na_454_00535**	4.281607	4.248144	3.945429	3.451869	3.665608	3.185161
Na_454_00535	4.168159	4.15134	3.891577	3.459607	3.668149	3.138147
Na_454_00535	4.092443	4.218219	3.942518	3.506567	3.684138	3.207264
Na_454_00535	4.187484	4.222936	3.907002	3.46879	3.609174	3.254287
Na_454_00535	4.23802	4.301236	4.034722	3.604563	3.804762	3.377464
Na_454_00535	4.176595	4.230007	3.952554	3.48177	3.73562	3.198989
Na_454_00535	4.239693	4.327848	4.109713	3.597071	3.712591	3.18303
Na_454_00535	4.263595	4.199302	3.948748	3.546799	3.709561	3.234582
Na_454_00535	4.169097	4.154273	3.887975	3.439	3.648739	3.251638
Na_454_00535	4.075606	4.103829	3.81862	3.365775	3.587223	3.104061
**Average**			4.129268			3.474998
**Standard Error (SE)**			0.026091			0.037872
**Na_454_00581**	7.096967	7.056708	7.211167	7.195171	7.015729	7.023168
Na_454_00581	6.567686	7.03442	7.210718	7.170819	7.114703	7.133321
Na_454_00581	7.247494	7.19532	7.364031	7.229319	7.123629	7.020622
Na_454_00581	6.956748	7.156795	7.361076	7.280362	7.211714	7.160087
Na_454_00581	6.661389	7.157372	7.400894	7.304877	7.104548	7.125672
Na_454_00581	7.063823	7.070447	7.285451	7.173761	7.092336	7.014684
Na_454_00581	7.115857	6.972707	7.211634	7.221645	7.019628	7.078234
Na_454_00581	7.082802	7.034516	7.291615	7.176515	7.127022	7.087216

Na_454_00581	7.021893	6.9349	7.213302	7.052899	7.048958	6.987499
Na_454_00581	7.154595	6.935175	7.226662	7.079099	7.076919	6.968235
**Average**			7.110286			7.122005
**Standard Error (SE)**			0.033718			0.015702

**Gene ID**	**WT-1**	**WT-2**	**WT-3**	**WT-4**	**WT-5**	**WT-6**
**Na_454_01047**	2.460512	2.823668	2.568578	2.40663	3.220239	2.298596
Na_454_01047	2.789143	2.798069	2.534196	2.4494	3.219415	2.342726
Na_454_01047	2.248332	2.822839	2.645724	2.49494	3.284437	2.426699
Na_454_01047	2.438708	2.812611	2.592268	2.39672	3.17658	2.289633
Na_454_01047	2.696269	2.793867	2.572603	2.446213	3.214271	2.351523
Na_454_01047	2.33276	2.850079	2.604688	2.565196	3.293225	2.408673
Na_454_01047	2.542775	2.818638	2.617767	2.440171	3.220189	2.436377
Na_454_01047	2.847686	2.799234	2.591878	2.40957	3.239919	2.324778
Na_454_01047	2.759175	2.855571	2.59303	2.396716	3.201236	2.280341
Na_454_01047	2.82215	2.841929	2.581069	2.416341	3.175982	2.332113
**Average**			2.659654			2.675349
**Standard Error (SE)**			0.030108			0.073434
**Na_454_01097**	-2.52688	-5.99075	-3.54886	-3.65025	-4.25997	-2.52725
Na_454_01097	-4.58409	-5.51163	-3.61002	-3.13261	-4.49203	-2.61749
Na_454_01097	-3.61644	-4.849	-3.82902	-3.79831	-5.18559	-2.59684
Na_454_01097	-2.36861	-5.12189	-3.67846	-3.84085	-4.37311	-2.56056
Na_454_01097	-2.68259	-4.51082	-4.02692	-3.97352	-4.59792	-2.58102
Na_454_01097	-3.84494	-5.04624	-3.88213	-3.69674	-4.79996	-2.47507
Na_454_01097	-2.56002	-5.52744	-3.83009	-4.17564	-5.08184	-2.81597
Na_454_01097	-2.58449	-5.19882	-4.42797	-3.8809	-5.75153	-2.68225
Na_454_01097	-2.82381	-6.19925	-3.98502	-3.68953	-3.92567	-2.65253
Na_454_01097	-4.56517	-5.40575	-3.62755	-3.25496	-5.23251	-2.29452
**Average**			-4.08764			-3.69685
**Standard Error (SE)**			0.195805			0.1785
**Na_454_01146**	4.571673	4.143926	4.154258	4.526415	3.729228	4.656185
Na_454_01146	4.534688	4.092646	4.143903	4.489918	3.822118	4.613841
Na_454_01146	4.423543	4.164838	4.207499	4.436543	3.786963	4.592306
Na_454_01146	4.636531	4.127133	4.20793	4.482807	3.8308	4.654004
Na_454_01146	4.635077	4.180717	4.231483	4.456441	3.809004	4.580966
Na_454_01146	4.643655	4.098947	4.177563	4.457651	3.814242	4.600925
Na_454_01146	4.693457	4.14598	4.237289	4.526942	3.740854	4.648768
Na_454_01146	3.524641	4.185071	4.239051	4.583367	3.777178	4.674045
Na_454_01146	4.483551	4.261571	4.311828	4.459782	3.725859	4.541771
Na_454_01146	4.352568	3.878053	4.102044	4.485846	3.696766	4.463381
**Average**			4.276239			4.296997
**Standard Error (SE)**			0.044816			0.068843
**Na_454_01281**	-2.25168	-2.66884	-2.59232	-2.414	-3.06631	-2.03691
Na_454_01281	-2.26808	-2.58197	-2.20681	-2.67035	-3.41827	-2.18412
Na_454_01281	-2.23086	-2.7474	-2.89237	-2.57928	-3.45529	-2.12667
Na_454_01281	-2.58187	-2.8462	-2.3937	-2.51466	-3.30986	-2.00721
Na_454_01281	-2.06089	-2.38963	-2.68264	-2.32881	-3.34543	-2.3582
Na_454_01281	-2.29065	-2.69579	-2.32553	-2.48598	-2.87309	-2.15816
Na_454_01281	-1.98527	-2.3126	-2.7137	-2.60603	-3.21074	-1.81528
Na_454_01281	-2.12513	-2.69567	-2.43993	-2.48574	-2.92737	-2.10422

Na_454_01281	-2.3266	-3.18431	-2.49758	-2.41131	-3.3154	-2.3763
Na_454_01281	-3.21668	-2.70157	-2.5273	-2.29156	-3.11953	-2.22982
**Average**			-2.48104			-2.61426
**Standard Error (SE)**			0.054634			0.087483

Gene ID	WT-1	WT-2	WT-3	WT-4	WT-5	WT-6
**Na_454_01409**	4.092009	3.704474	3.931586	3.567502	3.480359	3.505349
Na_454_01409	4.249029	3.856073	4.124197	3.775984	3.682702	3.713344
Na_454_01409	4.263742	3.830992	4.165333	3.750204	3.635446	3.698323
Na_454_01409	4.18281	3.842846	4.105311	3.755281	3.618756	3.713832
Na_454_01409	4.291477	3.862251	4.081456	3.779341	3.640211	3.713304
Na_454_01409	4.029095	3.706907	4.045529	3.821015	3.597455	3.66819
Na_454_01409	4.327555	3.775382	4.09946	3.7654	3.684239	3.591831
Na_454_01409	4.397808	3.932399	4.213247	3.835678	3.711505	3.755025
Na_454_01409	4.015012	3.756924	4.086043	3.721504	3.572092	3.694215
Na_454_01409	4.095032	3.746023	4.005091	3.606945	3.449448	3.546457
**Average**			4.035887			3.683263
**Standard Error (SE)**			0.035343			0.018014
**Na_454_01487**	5.258248	5.431272	5.447902	5.330085	5.272129	5.252139
Na_454_01487	5.437007	5.36252	5.445874	5.384118	5.218079	5.256125
Na_454_01487	5.467107	5.486246	5.54071	5.459965	5.402191	5.327166
Na_454_01487	5.451658	5.414402	5.521575	5.366114	5.276362	5.302348
Na_454_01487	5.608027	5.46316	5.557732	5.437122	5.366622	5.258097
Na_454_01487	5.306492	5.429015	5.551109	5.407357	5.352877	5.294707
Na_454_01487	5.633737	5.420533	5.512573	5.409169	5.426275	5.326679
Na_454_01487	5.299177	5.422491	5.480368	5.442231	5.5028	5.355873
Na_454_01487	5.35329	5.567183	5.666029	5.459826	5.440139	5.20086
Na_454_01487	5.377341	5.406601	5.424409	5.385984	5.366438	5.256891
**Average**			5.464275			5.352869
**Standard Error (SE)**			0.017543			0.014533
**Na_454_02132**	8.917799	8.784295	9.065522	8.846752	8.607615	8.791781
Na_454_02132	8.597234	8.711393	8.96537	8.793462	8.588431	8.765886
Na_454_02132	8.800393	8.785282	9.027667	8.873799	8.625196	8.758952
Na_454_02132	8.79539	8.711356	8.940885	8.713621	8.502344	8.693181
Na_454_02132	8.869356	8.734323	8.867989	8.741816	8.506238	8.738904
Na_454_02132	8.830055	8.758948	8.976766	8.833866	8.590118	8.784296
Na_454_02132	9.009479	8.728717	8.967468	8.801198	8.48605	8.774305
Na_454_02132	8.837213	8.804757	8.981824	8.764246	8.504642	8.804533
Na_454_02132	9.012829	8.842384	9.088994	8.879002	8.64042	8.794541
Na_454_02132	8.885026	8.708488	8.993275	8.927462	8.589121	8.855801
**Average**			8.867174			8.711304
**Standard Error (SE)**			0.022742			0.022918
**Na_454_02256**	5.601254	5.710153	5.917463	5.256379	4.661053	5.732323
Na_454_02256	5.40834	5.63682	5.888952	5.236946	4.684583	5.737542
Na_454_02256	5.463979	5.642051	5.882238	5.230486	4.698211	5.740149
Na_454_02256	4.523272	5.685784	5.890114	5.349827	4.669428	5.695332
Na_454_02256	5.670411	5.740783	5.936194	5.332818	4.712194	5.827104
Na_454_02256	4.55026	5.753787	5.934672	5.308732	4.673186	5.831124
Na_454_02256	5.630233	5.72129	5.971382	5.347345	4.745552	5.843958
Na_454_02256	5.800295	5.890774	6.053126	5.294044	4.758807	5.706878

Na_454_02256	5.716856	5.882675	5.941097	5.221236	4.669428	5.699362
Na_454_02256	5.465024	5.781942	5.922608	5.270523	4.735358	5.771657
**Average**			5.68312			5.246816
**Standard Error (SE)**			0.064164			0.080775

Gene ID	WT-1	WT-2	WT-3	WT-4	WT-5	WT-6
**Na_454_02680**	2.293291	2.233609	2.33469	2.225906	1.979177	2.023656
Na_454_02680	2.349614	2.135339	2.297692	2.189506	2.1006	2.125415
Na_454_02680	2.231426	2.239141	2.319298	2.165045	2.029161	2.141163
Na_454_02680	2.34267	2.373213	2.444317	2.426778	2.178529	2.313691
Na_454_02680	2.246356	2.284547	2.406147	2.254128	2.101432	2.164482
Na_454_02680	2.267329	2.282746	2.419923	2.322418	2.111842	2.183733
Na_454_02680	2.514714	2.342395	2.38753	2.268776	2.085528	2.206723
Na_454_02680	2.411568	2.305522	2.389296	2.246056	2.060738	2.202111
Na_454_02680	2.455883	2.330796	2.375307	2.246573	2.059011	2.199495
Na_454_02680	2.514216	2.391615	2.50176	2.190028	2.105398	2.204047
**Average**			2.333865			2.170803
**Standard Error (SE)**			0.016494			0.017807
**Na_454_03241**	5.672851	5.411571	5.626818	5.459555	5.279876	5.483634
Na_454_03241	5.765325	5.518574	5.755397	5.645014	5.360744	5.42552
Na_454_03241	5.709061	5.478278	5.751555	5.631449	5.394102	5.531151
Na_454_03241	5.687073	5.40763	5.634643	5.504594	5.300552	5.471008
Na_454_03241	5.683344	5.417931	5.706102	5.578046	5.360707	5.523514
Na_454_03241	5.6835	5.418494	5.680231	5.586759	5.375073	5.603659
Na_454_03241	5.717656	5.487061	5.732662	5.527889	5.398035	5.528451
Na_454_03241	5.386378	5.339992	5.587082	5.465181	5.416767	5.426993
Na_454_03241	5.45135	5.274641	5.454297	5.289161	5.171925	5.318979
Na_454_03241	5.574295	5.313988	5.65447	5.501085	5.257223	5.34962
**Average**			5.571833			5.446605
**Standard Error (SE)**			0.027344			0.021606
**Na_454_03693**	8.171557	8.939418	9.071892	8.954336	8.804364	9.039117
Na_454_03693	9.009503	8.959006	8.995144	8.868822	8.746171	8.870007
Na_454_03693	8.084452	8.97536	9.11396	8.948736	8.72032	8.926068
Na_454_03693	7.601394	8.953937	9.075366	8.903094	8.790295	8.881303
Na_454_03693	8.83071	8.87411	8.95588	8.869667	8.776174	8.809448
Na_454_03693	9.019256	8.914575	8.972596	8.921963	8.701422	8.854384
Na_454_03693	9.099228	8.962503	9.037519	8.857024	8.740127	8.829
Na_454_03693	9.033515	8.927	9.063487	8.891917	8.741186	8.867539
Na_454_03693	8.029856	8.902432	9.019191	8.977558	8.769775	8.912087
Na_454_03693	9.005658	8.895368	9.012777	8.931266	8.803107	8.954227
**Average**			8.836772			8.850811
**Standard Error (SE)**			0.066781			0.015409
**Na_454_04449**	1.246081	1.742701	1.803117	1.700862	1.546568	1.397079
Na_454_04449	1.86926	1.9104	1.887963	1.721979	1.599905	1.470345
Na_454_04449	1.078133	1.854387	1.837766	1.840864	1.633009	1.581403
Na_454_04449	1.77608	1.782298	1.702218	1.66833	1.529688	1.420868
Na_454_04449	1.693709	1.848874	1.866318	1.794865	1.575383	1.46639
Na_454_04449	1.766481	1.821751	1.85814	1.690427	1.597084	1.484146
Na_454_04449	1.762473	1.84613	1.765328	1.766262	1.650526	1.45259
Na_454_04449	1.606549	1.813	1.870152	1.783062	1.495597	1.456264

Na_454_04449	1.644556	1.779947	1.767379	1.712557	1.547611	1.420917
Na_454_04449	1.436618	1.767129	1.777275	1.768316	1.527319	1.507
**Average**			1.748192			1.592762
**Standard Error (SE)**			0.033737			0.023257

Gene ID	WT-1	WT-2	WT-3	WT-4	WT-5	WT-6
**Na_454_04958**	-5.92992	-5.98532	-6.16327	-6.47976	-5.66636	-6.61115
Na_454_04958	-4.53454	-6.14051	-6.33988	-6.65255	-6.59525	-6.46832
Na_454_04958	-5.9603	-6.08971	-6.13501	-6.59885	-6.54121	-5.58955
Na_454_04958	-6.18422	-6.19566	-5.9647	-6.70306	-6.64636	-6.52868
Na_454_04958	-6.17371	-6.24626	-6.43476	-6.76077	-6.67678	-6.93135
Na_454_04958	-5.20249	-6.27108	-5.32962	-6.80097	-5.66701	-6.99007
Na_454_04958	-6.09807	-6.1901	-6.35727	-6.67453	-6.64614	-6.86614
Na_454_04958	-6.06954	-6.22716	-6.43285	-6.73417	-5.59318	-6.91465
Na_454_04958	-6.0262	-6.16676	-5.10756	-6.64658	-6.57735	-5.76091
Na_454_04958	-5.7084	-5.80931	-5.9656	-6.21768	-6.28503	-6.40948
**Average**			-5.99839			-6.4934
**Standard Error (SE)**			0.077378			0.07488
**Na_454_05873**	-0.55907	-0.57514	-0.55444	0.343231	0.138595	0.448478
Na_454_05873	-0.70822	-0.58067	-0.68762	0.390747	0.245512	0.497685
Na_454_05873	-0.59212	-0.63992	-0.55066	0.296953	0.183067	0.414904
Na_454_05873	-0.56373	-0.63308	-0.66127	0.336249	0.155449	0.462091
Na_454_05873	-0.61941	-0.67629	-0.64517	0.281197	0.166639	0.494225
Na_454_05873	-1.04249	-0.56087	-0.53408	0.298543	0.274148	0.448179
Na_454_05873	-0.54959	-0.66971	-0.59419	0.36154	0.179069	0.477935
Na_454_05873	-0.64075	-0.62519	-0.60755	0.552491	0.253748	0.573195
Na_454_05873	-6.05282	-0.55596	-0.56784	0.357767	0.259982	0.598842
Na_454_05873	-0.48432	-0.57369	-0.31691	0.505824	0.386079	0.636755
**Average**			-0.82399			0.351499
**Standard Error (SE)**			0.182671			0.025512
**Na_454_09569**	-2.69496	-2.73586	-2.16006	-2.35324	-2.39077	-2.26006
Na_454_09569	-2.26128	-2.69234	-2.09054	-2.40385	-2.25665	-2.13751
Na_454_09569	-2.66148	-2.90997	-2.08268	-2.14412	-2.56001	-2.14195
Na_454_09569	-2.45578	-2.91106	-2.268	-2.45072	-2.58224	-2.25918
Na_454_09569	-2.41165	-2.71693	-2.42636	-2.53664	-2.78758	-2.32588
Na_454_09569	-2.44468	-2.52393	-2.28875	-2.41824	-2.54867	-2.1034
Na_454_09569	-2.48272	-2.91026	-2.29332	-2.35458	-2.66111	-2.30167
Na_454_09569	-2.35818	-2.59445	-2.20693	-2.40897	-2.80733	-2.1235
Na_454_09569	-2.34725	-2.60775	-2.14434	-2.68392	-2.67038	-2.23008
Na_454_09569	-2.45304	-2.80569	-2.41439	-2.41414	-2.77736	-2.29004
**Average**			-2.46969			-2.40379
**Standard Error (SE)**			0.04484			0.038118
**Na_454_11346**	2.997677	3.004299	2.935074	2.647098	2.919393	2.608629
Na_454_11346	2.216663	2.899045	2.961166	2.597582	3.018677	2.588171
Na_454_11346	3.406286	3.046887	3.087351	2.731723	3.110659	2.754458
Na_454_11346	3.318817	3.008265	3.050765	2.700213	3.149605	2.671003
Na_454_11346	2.338322	3.00864	3.051606	2.736108	3.16156	2.633806
Na_454_11346	3.576119	2.976466	3.069021	2.666917	3.087149	2.687211
Na_454_11346	3.342209	3.040084	3.006158	2.725632	3.099126	2.663497
Na_454_11346	3.227558	2.900011	2.91291	2.59154	3.062117	2.622083

Na_454_11346	3.294129	2.968948	3.026862	2.653697	2.968841	2.646048
Na_454_11346	3.303959	2.909596	2.979078	2.634703	3.016894	2.621154
**Average**			3.024864			2.79639
**Standard Error (SE)**			0.048411			0.036688

Gene ID	WT-1	WT-2	WT-3	WT-4	WT-5	WT-6
**Na_454_11519**	-5.91096	-6.03365	-6.24042	-5.66932	-6.48925	-6.67164
Na_454_11519	-3.79626	-6.17031	-6.39019	-6.69387	-6.63883	-6.87336
Na_454_11519	-6.13972	-6.28	-6.48609	-6.81346	-6.69943	-6.99124
Na_454_11519	-6.10733	-6.27286	-6.14321	-6.80943	-6.3146	-6.99772
Na_454_11519	-6.10057	-6.2682	-6.48302	-6.79914	-3.88612	-6.98894
Na_454_11519	-5.98238	-6.05441	-6.21755	-5.84675	-6.52895	-6.66744
Na_454_11519	-4.5775	-6.05993	-6.21512	-6.49998	-6.46622	-6.67158
Na_454_11519	-5.94193	-6.05961	-6.23675	-6.49919	-6.49354	-6.64308
Na_454_11519	-5.91261	-6.0247	-6.19734	-6.46088	-6.45935	-6.60105
Na_454_11519	-5.77674	-5.89479	-6.05187	-6.32279	-6.32626	-6.47568
**Average**			-6.01121			-6.48794
**Standard Error (SE)**			0.096837			0.103944
**Na_454_13228**	5.242868	5.187396	4.758692	5.435677	5.622139	5.465196
Na_454_13228	5.251841	5.172171	4.790378	5.477351	5.618313	5.519076
Na_454_13228	5.385828	5.210041	4.882698	5.580122	5.783157	5.552888
Na_454_13228	5.265976	5.135398	4.832311	5.552288	5.731008	5.554051
Na_454_13228	5.322724	5.141096	4.865966	5.542835	5.667053	5.564067
Na_454_13228	5.429273	5.218694	4.825903	5.530729	5.685403	5.507787
Na_454_13228	5.051061	5.177979	4.812734	5.505072	5.634892	5.571707
Na_454_13228	4.936936	5.248901	4.942274	5.605877	5.831672	5.651961
Na_454_13228	5.223774	5.157341	4.866179	5.489846	5.680121	5.568146
Na_454_13228	5.045598	5.140184	4.801236	5.516216	5.648449	5.507706
**Average**			5.086535			5.589942
**Standard Error (SE)**			0.036045			0.017105
**Na_454_14052**	1.166127	0.756634	0.699658	0.174946	0.482008	0.13525
Na_454_14052	1.383087	0.946884	0.812106	0.318267	0.704894	0.290419
Na_454_14052	1.126198	0.723689	0.733395	0.257514	0.616226	0.262938
Na_454_14052	1.222606	0.840878	0.79465	0.3035	0.578411	0.286124
Na_454_14052	1.275714	0.755746	0.735708	0.310159	0.671478	0.302745
Na_454_14052	1.22287	0.707566	0.792016	0.317872	0.548358	0.210531
Na_454_14052	1.310527	0.785533	0.816971	0.362789	0.552736	0.306067
Na_454_14052	1.262427	0.864672	0.821342	0.270667	0.597988	0.292351
Na_454_14052	1.445459	0.924754	0.813499	0.197101	0.614101	0.007843
Na_454_14052	1.073212	0.666032	0.655235	0.132519	0.429785	0.129262
**Average**			0.95336			0.369381
**Standard Error (SE)**			0.043944			0.033272
**Na_454_20559**	5.459991	5.18131	5.219769	5.613852	5.508891	5.64184
Na_454_20559	4.995404	5.140774	5.364242	5.560951	5.412828	5.629524
Na_454_20559	5.497836	5.291489	5.463633	5.78365	5.576529	5.804435
Na_454_20559	5.450382	5.294495	5.445929	5.758096	5.571478	5.826254
Na_454_20559	5.421386	5.257202	5.365087	5.751528	5.515007	5.737437
Na_454_20559	5.349078	5.205595	5.312526	5.62722	5.477323	5.674453
Na_454_20559	5.445868	5.213919	5.32684	5.640053	5.461629	5.664702
Na_454_20559	5.477423	5.324095	5.496439	5.822252	5.622775	5.775914

Na_454_20559	5.247261	5.018524	5.153608	5.510977	5.382131	5.506886
Na_454_20559	5.389252	5.18074	5.309569	5.651788	5.506736	5.668936
**Average**			5.311856			5.624393
**Standard Error (SE)**			0.02476			0.022526

**Gene ID**	**WT-1**	**WT-2**	**WT-3**	**WT-4**	**WT-5**	**WT-6**
**Na_454_29718**	-2.01712	-1.91554	-1.98863	-2.19373	-2.02003	-2.3127
Na_454_29718	-2.01147	-1.91141	-2.04179	-1.96599	-2.10744	-2.05293
Na_454_29718	-2.09636	-1.87702	-1.82825	-2.08144	-2.02184	-1.8663
Na_454_29718	-1.98012	-1.87473	-1.76793	-2.04098	-2.06073	-2.04856
Na_454_29718	-1.76593	-2.03127	-2.05671	-2.03669	-2.13128	-2.05982
Na_454_29718	-1.78806	-1.85875	-2.00739	-2.09818	-1.8383	-2.16145
Na_454_29718	-2.0935	-1.94545	-1.78749	-2.01187	-1.8946	-1.9988
Na_454_29718	-1.79572	-1.89871	-1.77559	-1.82731	-2.1495	-1.95
Na_454_29718	-2.04509	-2.31069	-2.09413	-2.06113	-2.14344	-1.97073
Na_454_29718	-2.98248	-2.1434	-2.07266	-2.0585	-2.17605	-2.05451
**Average**			-1.94685			-2.04095
**Standard Error (SE)**			0.041812			0.019522
**Na_454_36014**	-4.16016	-4.39376	-4.00275	-4.50372	-3.90452	-4.77664
Na_454_36014	-4.11649	-4.26969	-4.21351	-4.54552	-4.08465	-6.66695
Na_454_36014	-5.83947	-4.01508	-4.22811	-4.12755	-4.94371	-6.56377
Na_454_36014	-4.32903	-4.13961	-4.7443	-3.97902	-4.27062	-4.64266
Na_454_36014	-4.05067	-4.28772	-4.24122	-4.41347	-4.72066	-5.30482
Na_454_36014	-4.87003	-4.064	-5.08805	-3.85287	-4.17587	-5.1166
Na_454_36014	-4.16184	-4.2166	-4.09488	-4.07305	-4.09592	-5.39502
Na_454_36014	-4.51573	-4.08478	-4.43798	-4.28143	-4.43134	-4.77271
Na_454_36014	-4.70159	-3.69352	-5.27064	-4.20797	-5.19283	-5.41014
Na_454_36014	-5.80553	-4.2766	-4.02116	-4.11403	-4.53781	-5.90241
**Average**			-4.37893			-4.68348
**Standard Error (SE)**			0.093046			0.133173
