# Supplementary material for: A Jasmonate ZIM-Domain Protein NaJAZd Regulates Floral Jasmonic Acid Levels and Counteracts Flower Abscission in Nicotiana attenuata Plants
Source: PLoS One. 2013 Feb 28;8(2):e57868. doi: 10.1371/journal.pone.0057868 (PMC3585257; doi:10.1371/journal.pone.0057868)
Supplement: Text S1 — Nucleotide sequence alignment of NaJAZ genes and inverted repeat (ir) construct used for NaJAZd silencing in irJAZh plants. (PDF) [file pone.0057868.s012.pdf]

Text S1. Nucleotide sequence alignment of *NaJAZ* genes and inverted repeat (ir) construct used for NaJAZd silencing in irJAZh plants.

A. Sequence alignment of NaJAZa and ir-construct of NaJAZd

```
ir-construct      ------------------------------------------------------------
NaJAZa            ATGGCATCATCGGAGATTGTGGATTCCGGTAAATTTTCCGCCGGCGGCGGTCAGAAATCA 60

ir-construct      ------------------------------------------------------------
NaJAZa            CATTTTTCACAGACATGTAATTTGTTGAGCCAATACTTGAAAGAGAAGAAAGGTTCCTTT 120

ir-construct      ------------------------------------------------------------
NaJAZa            GGAGATCTCAGCCTTGGTATCCACCGCGCCGGCACTACTACTATGGATTTATTCCCAATG 180

ir-construct      ------------------------------------------------------ACTA-- 4
                                                                        ***
NaJAZa            ATTGAGAAATCTGGTGAGTCAAACCCTCAGAAACCAATGAATCTGTTTCCTCAAACTGAA 240

ir-construct      -CAAAATCTGAACCAGAAAAGGCACAAATGACAATATTTTATGGTGGTCAAGTTATTGTA 63
                   *********** * ** ** ***** ***** ***** ** ** ***************
NaJAZa            GCAAAATCTGAATCGGAGAAAGCACAGATGACGATATTCTACGGCGGTCAAGTTATTGTA 300

ir-construct      TTTGATGATTTTCCAGCAGCTAAGGCAAATGAAATCATGAAATTGGCTAG------CAAG 117
                  *** ********** **** *** ** ** *********    *******      ***
NaJAZa            TTTAATGATTTTCCGGCAGATAAAGCTAAGGAAATCATGCTTATGGCTAGTTGTGCCAAA 360

ir-construct      AAAAACAACAACAA-------------CAACAAACAG---AATTTGGCTACTAACATATT 161
                    ***********             *** ******   *** ** **    *  *  *
NaJAZa            GGAAACAACAACAGTACTACTCAGATTCAAAAAACAGCTGAATCTG-CTTTAGATTTGGT 419

ir-construct      TTCTTATCCTAT-------GGTAAATAATCAAAATTCAGCTGAATCTG--TTACTACCAA 212
                    ** * *****       **  * * * ***     **  **   *   ******
NaJAZa            ACCTCAGCCTATTATTTCTGGAGATTTACCAATTGCGAGACGAGCTTCACTTACTAGGTT 479

ir-construct      TTT-GACTCAAGAACT------TCGTTCGCGAACTCAC-GTGCCAAT--ATCA------- 255
                  *** **   *****          * * ** **  *** ** *****  * **
NaJAZa            TTTAGAGAAAAGAAAAGATAGGCTGATTGCTAAAGCACCGTACCAATTAAGCAACACAAA 539

ir-construct      ----CAATCATCTGTTGCTGA-------------TTTA----------CCGATTGCGAGA 288
                      *** ** * *** ****              **            * *** * **
NaJAZa            TAAACAAGCAGCAGTTTCTGAAAACAAGGCGTGGCTTGGATTGGGTGCTCAATTTCCAGT 599

ir-construct      CGAAATT---CACTTACA- 303
                    **  *   ** **  *
NaJAZa            GAAAGCTGAGCAATTCTAG 618
```

B. Sequence alignment of NaJAZb and ir-construct of NaJAZd

```
ir-construct      ----------------------------------ACTACAA-----AATCTGAACCAGAA 21
                                                    **  ***      *  ** ** *****
NaJAZb            ATGGATTCAAGTATTATTGAGATAGATTTCATGGACCTCAACAGCAGACCTCAATCAGAA 60
```

```
ir-construct      AAGGC------ACAAA--------------------TGACAATATTT---TATGG----- 47
                  * ***      *****                    ** * ** **    *****
NaJAZb            ATGGCGAAGCAACAAACCAAAGCTTCTGGGATGAAGTGGCCATTTTCATCTATGGCTAAC 120

ir-construct      -----TGGTCAAGTT--------ATTGTATTTGA---------------TGAT--TTTCC 77
                       *  ***** *        *** * *  **                 **  *****
NaJAZb            TTAGCTACTCAAGCTGAAAGCAGATTTTTTCAGAATTACAACTCCTCCCCAATAGTTTCC 180

ir-construct      AGCAG----------------CTAAGG----------------CAAATGAAATCATGAAA 105
                  ** *                 **** *                **** **   ** ****
NaJAZb            AGTAATTCAAAAAGTTCACCCCTAAAGAGCTACAAACTCACCACAAAAGATTCCAGGAAA 240

ir-construct      ----TTGGCTAGCAAGAAAAAC-------AACAACAAC---------------------- 132
                       ** * *       ****       ***    **
NaJAZb            AATTCTGACCATTTGAGGAAACCTGAGTCAACGTTGACCATATTCTACATGGGTGAGGTC 300

ir-construct      ----------------------------------AACAAACA--GAATTTGGCTACTAA- 155
                                                    *** ** *  * * ****** ****
NaJAZb            CATATTTTTCAGGATATCACACCAGAAAAGGCTGAACTAATAATGGACTTGGCTTCTAAA 360

ir-construct      ----CATATTTTCTTATC-CT------------ATGGTAAATAATCAAAATTC------A 192
                      *  ** * *  **  **            * ** ******  **** *       *
NaJAZb            TCAACGAATCTCCACATGACTGAGATTTTAGAAAAGGCAAATAAAGAAAAATATGAAGAA 420

ir-construct      GCTGAATCTG-----TTACTACCAATTTGACTCAAGAACTTCGTT-------CGCGAACT 240
                    * ******     * *  ***** *  *  **  ** *  * *       * *   **
NaJAZb            AATAAATCTGAGCCTTCAACACCAAATGCATCCACAAATTATGCTAAAGGAGCACTGGCT 480

ir-construct      ----CACGTG-------------CCAAT--------------------------ATCACA 257
                      * ***              ** **                          ******
NaJAZb            ATGGCTCGTAGAGCAACCCTTGCCCGATTTTTGGAGAAGAGAAAGCATAGATTGATCACA 540

ir-construct      ATCATCTGTTGCTGATTT-----------ACCGA---------TTG--------CGAGAC 289
                     *     * *  ** *           *** *         ***        * ***
NaJAZb            GCTAGGCCATACCAATATGGTGAAAAAACACCAAAGTTTCCTTTTGAAATGCACCAAGAA 600

ir-construct      GAAA------------------TTCACTTACA------- 303
                  ***                   **** *   *
NaJAZb            GAAGAAACGGCGTCGTCAAGCGTTCATTGGGAAAGCTAA 639
```

C. Sequence alignment of NaJAZc.1 and ir-construct of NaJAZd

```
ir-construct      ------------------------------------------------------------
NaJAZc.1          ATGGAGAGAGATTTTATGGGACTGAATTCTAAAGATTCTGTTGTTTTGGTTAAGGAAGAA 60


ir-construct      ------------------------------------------------------------
NaJAZc.1          CCTGTTGAAACCTGCAAAGACTCTGGGTTTCGCTGGCCGTTGTCGAGCAAGGTGGGTATA 120


ir-construct      ------------------------------------------------------------
NaJAZc.1          CCTCATTTCATGTCTTTGAACTCTGCTCAAGATGAAAAACCATTCAAAGCTCAATCCGCT 180


ir-construct      ------------------------------------------------------------
NaJAZc.1          GCAGATGGAGTTGACAGTTGTCTCAAACGCCAGTCTGGTGAAATCCAGAATGTGCATGCA 240


ir-construct      ------------------------------------------------------------
NaJAZc.1          ATGCATCTTCTCCATGATGTTATGATGCTTCCGTTTAACAGGAGCAATCCCTCCTACAAG 300
```

```
ir-construct      ------------------------------------------------------------
NaJAZc.1          ACTCATTTTGGTGGTACTGGCCAGATATCTGCAGCTGCGACAATGAAGCAGCAACTTCTT 360

ir-construct      ------------------------------------------------------ACTACA 6
                                                                         **
NaJAZc.1          GGGGGAATCCCTGTTACAGCTCCTCACTCAATACTTCCATCGAGTGGCTCTGTGGCTGGG 420

ir-construct      AAATCTGAACC--AG---------AAAAG-------------GCACAAATGACAATATTT 42
                  * * *******   *         *** *             ** *** **** ** **
NaJAZc.1          ATAACTGAACCTTGGTTTAATTCCAAAGGTTCTGCAGCACCTGCTCAACTGACCATCTTC 480

ir-construct      TATGGTGGTCAAGTTATTGTATTTGATGATTTTCCAGCAGCTAAGGCAAATGAAATCATG 102
                  ********   *** * *** ********* *  *  * *  ****** * *  ** ***
NaJAZc.1          TATGGTGGAACAGTCAATGTCTTTGATGATATCTCCCCTGAGAAGGCACAGGCTATTATG 540

ir-construct      AAATTGGCTAG--------------------------------CAAG-----AA----AA 121
                     ****** *                                ****     **    *
NaJAZc.1          TTTTTGGCTGGAAATGGTTGTGTTCCTCCTAATGTGGTGCAACCAAGGTTTCAAGTTCAG 600

ir-construct      ACAACAACAAC--AAC----------AAACAGAATTTG----------GCTACTAACATA 159
                   ** * *** * ***          * ** * *  **           *  * ******
NaJAZc.1          GCATCTACACCCAAACTTGCTGCCGTAGACGGCACCTGTGTGAACCAAACCTCAAACATA 660

ir-construct      TT------------------------------------TTCTTATCCTAT-GGTAAAT-- 180
                   *                                    **** **** ** *** ***
NaJAZc.1          CTACCTGCCTCAGGTCATTCCAGCCCTATGTCTGTTTCTTCTCATCCCATTGGTCAATCT 720

ir-construct      --------------AATCAAAATTCAGCTGAA----TCTGTTACTACCAA--TTT----- 215
                                ** ****  * *  ****    ***   *** * **  ***
NaJAZc.1          GCTGGCAATTCTGGAAACAAAGATGACATGAAGATATCTAAAACTGCAAACATTTCAGTG 780

ir-construct      --GACTCAAGAACTT-----------------CGTTCGCGAACTCAC----GTGCCAATA 252
                    ***** * *  **                  *** * *     **     ** **  *
NaJAZc.1          GAGACTCCAAAGATTATGACATCACTAGGACCTGTTGGGGCTTCTACCATAATGTCAGCA 840

ir-construct      ------TCACA---------ATCATCTGTTGCT-GATTT--------------------- 275
                         ****         * ***** * *** * ***
NaJAZc.1          GCGGTTCCACAGGCAAGGAAAGCATCTCTGGCTCGGTTTCTGGAGAAGCGGAAGGAAAGG 900

ir-construct      --------------ACC-------------------------GATTGC---------GAG 287
                                ***                         ** ***         ***
NaJAZc.1          GTGATGAACGCTGCACCATATGGCCTCAGCAAGAAATCGGGTGAGTGCTCCACCCCAGAG 960

ir-construct      ----ACGAAATTC-----------ACTTACA----------------------------- 303
                      *** * **            **** **
NaJAZc.1          TCTAACGGAGTTGGTTTCTCTGCAACTTCCAGTGTTGGTACTAGTCCTCTGATAGCCGGT 1020

ir-construct      ------------
NaJAZc.1          AAGGAGACCTAG 1032
```

D. Sequence alignment of NaJAZe and ir-construct of NaJAZd

```
ir-construct      ---------ACTACAAAATCTGAACCAGAAA-------AGGCACA--------------- 29
                           *** **  ** **** **  **       * * ***
NaJAZe            ATGGGTTTGACT-CATCATGTGAAGCAAGAAGTAATTGAAGAACATATAGATCCAGCACC 59

ir-construct      ---------------AATG----------------ACAATATTTT--------------- 43
                                 ****                **** ** *
NaJAZe            TCTGAGAAGTTCAGCAATGCAGTGGTCATTCTCGAACAACATCTCGACTCATCCTCAATA 119

ir-construct      -----------ATGGTGGTCAAG--------TT----ATTGTATTTGATGATTTTCCAGC 80
                             * **** *****         *    * **  ******    ** ** *
```

```
NaJAZe            CCTCTCTTTCAAGGGTGCTCAAGAGGATAGGCTGAAAACTGGTTTTGATTCACTTGCATC 179

ir-construct      AGCTAA---GGCAAATGAAATCA----TGAAATTG---------GCTAGCAA-------- 116
                  * **     **  * *  ** **    ****  **         *  * * *
NaJAZe            AACTGGATTGGTGACTATAACCACAACTGAAGCTGTCGACTCAAGTCATCGACCGTACTC 239

ir-construct      ----------------------------GAAAAACAA-------------------CAAC 129
                                              ***** ***                   * **
NaJAZe            TGGTGTCACACAGAATAATATGATGCTTGAAAAGCAAGGTGGAACGCACTACACGTCGAC 299

ir-construct      AAC--------------------------------------------------------- 132
                  ***
NaJAZe            AACTTTCTCTCCTCATCACTATGATGCTCACTCCGTGCATCGATCTCATGGAGTCAGAGT 359

ir-construct      ------------------AACAAA-CAGAATT---------TGGCTA---CTAA-CATA- 159
                                    * **** **** **         ** ***   **   ****
NaJAZe            GCTCCCAGTTTCCAACCTAGCAAATCAGATTTCTGTATCTATGACTATGCCTGGTCATAA 419

ir-construct      ----TTTTCTTATCCTATGGTAAATAATCAA------------AATTCAGCTG----AAT 199
                      ***  ** **** * * * * **** *            * ********    **
NaJAZe            GTCCTTTGTTTCTCCTCTTGGACAGAATCCAGTTGCTAGCCCCATTTCAGCTGTTCCAAC 479

ir-construct      --------CTGTT------ACTACCAATTTGA-------CTCAAGAACTTC--------- 229
                          ****       ** **  **** *         * * **** *
NaJAZe            TAACAGCGCTGTCGTGGGCACAACTGATTTAAGGGGTGCTCCGAAAACTCCCCCAGGTCC 539

ir-construct      -------------------------------GTTCGCG---------------------- 236
                                                 **  * *
NaJAZe            TGCTCAGTTGACCATCTTTTATGGTGGTTCCGTCTGTGTTTATGATAATGTTTCGCCAGA 599

ir-construct      ------------------------------------------------------------
NaJAZe            GAAGGCTCAAGCTATTATGTTGCTTGCTGGAAATGCACCACCTGTTACACCAAGTGCAAC 659

ir-construct      ---------------AACTCACGTGCCAATATCACAATCATCTGTTGCTGA----TTTAC 277
                                 *  *** * *** *** *  * ** ***  ** ***    ***
NaJAZe            ATCTACTCTATCTCCAGTTCAGGCGCCTATACCCAAGTCCTCTTCTGTTGACTCTTTTGT 719

ir-construct      CG---------------------------------------------------------A 280
                   *                                                         *
NaJAZe            TGTAAATCAGTCCCACAACACAACACCTACTCTTCCCAGCCCCATTTCTATAACATCTCA 779

ir-construct      TTGCGAGACGAAATTCACT--------------TACA----------------------- 303
                  *** *   *  ***   **              ****
NaJAZe            TTGTGGATCTCAATCTGCTGGAGTGTCTAGTAATACAAATGGAGTAACTATTATCAAATC 839

ir-construct      ------------------------------------------------------------
NaJAZe            AACTGGGGTCCTACCATCTCCTTCTAATAAAGCAGGACTTTCCAAATTTTCCAGTTCCAT 899

ir-construct      ------------------------------------------------------------
NaJAZe            AGGATCTGTTCCTGCCACCTTTGTTCCATCAGCTGTACCGCAGGCACGCAAGGCATCATT 959

ir-construct      ------------------------------------------------------------
NaJAZe            GGCTCGGTTCTTGGAGAAGCGCAAAGAAAGGGTAATAAGTGCATCACCTTACGACACCAG 1019

ir-construct      ------------------------------------------------------------
NaJAZe            CAAGCAATCCCCAGAATGTAGCACTCTTGGATATGGAAGCAGAAGTTTCGCTAAATATTC 1079

ir-construct      -------------------------------------------------
NaJAZe            TTTAGGCTCTTGTCCTCCCCAAGTAATCAATTTGGTCAAGGAGACGTGA 1128
```

E. Sequence alignment of NaJAZf and ir-construct of NaJAZd

```
ir-construct      ---ACTACAAAATCTGAACCAGAA--AAGGCACAAATGACAATAT-TTTATGGTGGTC-- 52
                     *  * *** * * *    ***  *****        *  * * *** ** *  **
NaJAZf            ATGAGAAGAAACTGTAACTTGGAACTAAGGCTTGTCCCTCCTTGTGTTTCTGTTTCTCCT 60

ir-construct      -AAGTTATTGTATTTGATGATTTTCCAGCAGCTAAGGCAAATGAAATCATGAAATTGGCT 111
                   ***  **** * *  * **      *    **    **   *  ** *  *    ***
NaJAZf            AAAG--ATTGCACT--ACGACCCCTTA----CTTCTCCATGAGGGATAACCAG---GGCA 109

ir-construct      AGCAAGAAAAACAACAACAACAACAAACAGAATTTGGCTACTAACATATT-----TTCTT 166
                     **** ** ***** ** ** * ****  ***   * *   * *  **     ****
NaJAZf            CAGAAGAGAAGCAACAGCAGCAGCTAACAATATTCTACAATGGAAAAGTTGTGGTTTCTG 169

ir-construct      ATCCTATGGTA--------------AATAAT--------CAA-------AATTCAGCTGA 197
                  ** ***  *                ******        ***       **   **  **
NaJAZf            ATGCTACAGAGCTTCAGGCGAAAGCAATAATATATCTCGCAAGTAGAGAAACGGAGGAGA 229

ir-construct      ATCTG------TTACTACCAATTT--GACTCAAGAACTTCGTT--CGCGAACTCACG--- 244
                  **         * *  ********  ** ***  * *  * **    * ******
NaJAZf            ATACAAAGACTTCATCACCAATTTCAGAATCATCATCACCATTGTTACAAACTCAAACTG 289

ir-construct      ---TGCCAATATCACAATCATCTGTTGCTGATTTACCGATTGCGAGACGA---AATTCA- 297
                     *  * **     **** ****       **   * *  * ** *  *   ******
NaJAZf            GTCTTTCCATGAAGAAATC-TCTGCAAAGATTTCTGCAAAAGAGAAAAAATAGAATTCAA 348

ir-construct      ----CTT----ACA---------- 303
                      ***    * *
NaJAZf            GAAACTTCTCCATATCATCACTAG 372
```

F. Sequence alignment of NaJAZg and ir-construct of NaJAZd

```
ir-construct      ------------------------ACTACAAAAT---------CTGAA------------ 15
                                           **  ****          *****
NaJAZg            ATGGAGAGAGATTTCATGGGGTTGGCTGTAAAACAAGAAATACCTGAAGAACAGCCCACA 60

ir-construct      ---CCAGAAAAGGCACAAAT---GACAATATT-------TTAT----------------- 45
                     **** ** ***   ***   * * *****       * **
NaJAZg            GATCCAGCAATGGCCAGAATTTCGGCTATATTGCAGAGGTCATTCTCTAACAAGGCTCTT 120

ir-construct      ---------------------GGTGGTCAAG------TT------ATTGTATTTGATGAT 72
                                         ** *****       *      * **  ******  *
NaJAZg            CCTCAATACCTTTCTTTCAAAAATGCTCAAGGGAATACTCCTAAGACTGGTTTTGATTCT 180

ir-construct      TTTCCAGCAGCTAA---GGCAAATG-------AAATCATGAA----------------AT 106
                   ** ** *****     **  * *        ** *******                **
NaJAZg            CTTGCATCAGCTGGATTGGTTACTATAACCACAAGTCATGAAGCTGTTGACTCGAATTAT 240

ir-construct      TGGCTA---------GCAA--------------------GAAAAACAA------------ 125
                   * * *         * **                    ***** ***
NaJAZg            CGACCATACACTGCTGTAACACAGAAAAATTTGATGCTTGAAAAGCAAGGGATAACGAAC 300

ir-construct      ----CAACAACAAC---------AAACAGAATTTG------------------------- 147
                      ***  *****         * * * **** *
NaJAZg            TATACAATGACAACTTACCCTCCACATAAAATTGGTACAAATTCAGTTCAGCAATCTCAT 360

ir-construct      ------------------GCTACTAA----------CATATTTTCTTATCC--------- 170
```

```
                                      * * ****          ** ****   ****
NaJAZg            GAAGTCAGAGTACTCCCAGTTGCTAATCAAACACATCAGATTTCTGTATCTACGAGAAAT 420


ir-construct      ---TATGGT-----------------------------AAAT---------------AAT 183
                      *****                              ***               ***
NaJAZg            ATGCATGGTCGTCAGCCCTTAATTTCTCCTGCTGGACAGAATTTGATTTCTATAATAAAT 480

ir-construct      CAAAATTCAGCTGAA------------------TCTGTT--------------------- 204
                  ****** * ***  *                  **  **
NaJAZg            CAAAATCCTGCTAGAGGTGCCCAAATATCAAGCTCCATTTCCATTCTTCCTAATCGCAAT 540

ir-construct      ------------ACTACCAATTTGA--------CTCAAGA--------------ACTTCG 230
                              *****  * ****        * *****               **  *
NaJAZg            GGAGTGGTGGGCACTACTGAATTGAGGGGTGCTCCCAAGACTTCAGCAGGACCTGCTCAG 600

ir-construct      TTCGCGAACTCACGTGC---------------------CAATAT---------------- 253
                   *  * * **    ***                     ******
NaJAZg            CTGACCATCTTTTATGCTGGTTCAGTCAGTGTTTACGACAATATTTCTCCAGAGAAGGCT 660

ir-construct      ----------------------------CACAATCATCTG------------------TT 267
                                              ***** ** ***                   *
NaJAZg            CAAGCTATCATGTTACTTGCTGGCAATGCACAACCAGCTGGTATTCCAAGTACAACATCT 720

ir-construct      GCTGATTTACCGATTGCGAGA----CGAAATTCACTT---------------------AC 302
                   ***  *  **  **  ****    * ****   ***                     **
NaJAZg            ACTGCATCTCCAGTTCAGAGAATTCCTAAATCTTCTTCTGTTGATGCTTTTGTTGGAAAC 780

ir-construct      A----------------------------------------------------------- 303
                  *
NaJAZg            AAATGCCATAGAACTACATCGCCTAGTTTTTCCAGTCCCATTCCTATAACCACACACGGC 840

ir-construct      ------------------------------------------------------------
NaJAZg            GCCTCTCAATCTATTGGAGTGTCTAATAATACGAATCAAATAACTATGAGTATCAGGTCA 900


ir-construct      ------------------------------------------------------------
NaJAZg            ATCGGAGTCCTGACTAATTCTCCCTCTAACAAAACGGAGCCATCTAAAGTCGTCCGTTCT 960


ir-construct      ------------------------------------------------------------
NaJAZg            CAGGAATCTCATCCTCCTAGCCATACATTATCAGCTGTACCTCAGGCTCGCAAAGCATCC 1020


ir-construct      ------------------------------------------------------------
NaJAZg            TTGGCTCGGTTCTTGGAGAAGCGCAAGGAAAGGATACTGAGTGCATCACCATACGACAAT 1080


ir-construct      ------------------------------------------------------------
NaJAZg            AGCAAGCAAAGTTCACAATATAGTACACCTGGATCTAGCAGCTGGAGCTTCTTTGTTAAC 1140


ir-construct      ---------------------------------------
NaJAZg            TCTTCAGGATCCAGTACTGTTCTTCCTGCTACTATATAG 1179
```

G. Sequence alignment of NaJAZh and ir-construct of NaJAZd

```
ir-construct      ------------------------------------------------------------
NaJAZh            ATGTCAAATTCGCAAAATTCTTTTGACGGCGGCAGAAGGGCCGGAAAAGCGCCGGAGAGA 60


ir-construct      ------------------------------------------------------------
```

```
NaJAZh            TCGAATTTCGTGCAGACTTGTAATTTATTGAGTCAGTTTATTAAAGGAAAAGCTACTATT 120


ir-construct      ------------------------------------------------------------
NaJAZh            AGAGATCTGAATCTCGGAATTGCTGGAAAATCTGAAATCTCAGGTAAAAGTGATGTTACA 180

ir-construct      ------------------------------------------------------------
NaJAZh            GAAGCTGCAACTATGGATTTATTGACAATTATGGAAAACCCCTCAATTGAAACTAAAGAA 240

ir-construct      ------------------------------------ACT---ACAAAATCT--------- 12
                                                       **   *** *****
NaJAZh            CAAGAACAAAAATCCATAGATCCCGTTCGTCAGAGTGCTGTAACAGAATCTTCTAGAGAT 300

ir-construct      ---------------------------------------GAACCAGAAAAG--------- 24
                                                         * **** ** **
NaJAZh            ATGGAGGTGGCCGTAAATGAGCCCAGCACGAGCAAAGAGGCACCAAAAGAGCCTAAGGCA 360

ir-construct      GCACAAATGACAATATTTTATGGTGGTCAAGTTATTGTATTTGATGATTTTCCAGCAGCT 84
                  ****** **** ** ** **** **** **** ** ******************** *
NaJAZh            GCACAATTGACTATGTTCTATGATGGTAAAGTGATAGTATTTGATGATTTTCCAGCTGAC 420

ir-construct      AAGGCAAATGAAATCATGAAATTGGCTAGCAAGAAA------------------AACA-- 124
                  ** ** *  * * * ***  ********* **   *                    **
NaJAZh            AAAGCTAGAGCAGTAATGTTATTGGCTAGTAAAGGATGCCCTCAGAGTTCATTTGGCACT 480

ir-construct      ------ACAACAACAACAAACAGAATTTG-------GCTACTA----------------- 154
                        ** ***** *   *** ****         ******
NaJAZh            TTTCATACTACAACCATCGACAAAATTAACACATCTGCTACTGCTGCTGCCACAGCTTCT 540

ir-construct      ---ACATAT-------------------------------TTTCT--------------- 165
                     **** *                               *****
NaJAZh            TTGACATGTAATAAAACTAATCAGCTTAAACCAAGTACAGTTTCTATTGCACCACCACAA 600

ir-construct      ----------------------TATCCTATGGTAAA--TAATCAA--------------- 186
                                        * ** *** *****  * * ***
NaJAZh            CAAAAGCAGCAGCAAATTCATGTTTCTTATAGTAAAAGTGACCAACTCAAGCCAGGGTAT 660

ir-construct      AATTCAGCTG-------------------------AATCTGT-----TACTACCAAT--- 213
                  ***** ***                          * **  *     ** *** * *
NaJAZh            AATTCTGCTACGCCGCAAGTACTGCAGCAGCAGCTAGTCCATGTTTCTAGTACTAGTAAA 720

ir-construct      -----TTGACTCAAG--------AACTTCGTTCGCG-------AACT-CACGTGC----- 247
                       *   ** ***        ****** *  ***       ***  ** * **
NaJAZh            ACTGATCAGCTTAAGCCAGTATCAACTTCTTCTGCGTCGCAAAAACAGCAGGAGCAACAT 780

ir-construct      CAATA-------TCACAATCATCTG----TTGC-----TGATTTACCGATTGCGAGACGA 291
                  **  *       *****  ** ***    * **     ***  **** ***** *** **
NaJAZh            CAGCAAACGCAGTCACAGACACCTGGAACTAGCAGCTCTGAGCTACCTATTGCAAGAAGA 840

ir-construct      AATTCACT---------------------------------------------------- 299
                     *****
NaJAZh            TCATCACTACATAGGTTTCTTGAGAAGAGGAAAGATAGGGCAACGGCTAGAGCGCCATAC 900

ir-construct      -------TACA------------------------------------------------- 303
                         ****
NaJAZh            CAAGTTGTACATAATAATCCGTTACCATCATCTTCAAATAATAATGGGGAATCATCTTCC 960

ir-construct      ------------------------------------------
NaJAZh            AAGGATTGCGAAGATCAACTCGATCTCAATTTCAAGTTATAG 1002
```

H. Sequence alignment of NaJAZj and ir-construct of NaJAZd

```
ir-construct      ACTACAAAAT-CTG-AACCAGAAA---AGGCACAAATGACAATATTTTA-TGGTGGTC-- 52
                  *  * ****  *** ***  * *    ****  *     *  * ****  *  *  **
NaJAZj            ATGAGAAAAAACTGTAACTTGGAGCTCAGGCTTATGCCTCCTTCTTTTTCTTTTTCTCCT 60

ir-construct      --AAGTTATTGTATTTGATGATTTTCCAGCAGCTAAGGCAAATGAAATCATGAAATTGGC 110
                     ****    **     *  ** ** *     ** **   ** **     ****
NaJAZj            AAGAGTTGCACTACCCCCTACTTCTCTA-TGAATAGGGAGGATAAA-----GAAAACA-- 112

ir-construct      TAGCAAGAAAAACAACAACAACAACAAACAGAATTTGGCTACTAACA-----TATTTTCT 165
                   ** **** *** *** *** ** * ****  ****  * *   * *     *  *****
NaJAZj            CAG-AAGAGAAAGAACCACAGCAGCTAACAATATTTTACAATGGAAAGCTTGTGGTTTCT 171

ir-construct      TATCCTA------------------------------TGGTAAATA-----ATCAAAATT 190
                   ** ***                              *** ** **     **  * *
NaJAZj            GATGCTACTGAACTTCAGGCTAAAGCAATAATATATCTGGCAAGTAGAGAAATGGAGAAG 231

ir-construct      CAGCTGAATCTGTTACTACCAATTT--GACTCAAGAACTTCGTTCGC-GAACTCACGT-G 246
                   *  * **  *      ********  ** ***  * *  *  *  * ** *   * *
NaJAZj            AAAATAAAAATCCGGTCACCAATTTCAGAATCATCATCACCAATTTCAGAGCCTTCATCA 291

ir-construct      CCAATATCACAATCATCTGTTGCTGATTTACCGAT-----------------------TG 283
                  *** * * ******  * * * ***** *  * **                       **
NaJAZj            CCACTTTTACAATCTCCAGCTTCTGATATTTCTATGAAGAGATCTCTACAAAGGTTTCTG 351

ir-construct      CGAGA---------CGAAATTCA-----------CTTA-CA------- 303
                  * * *            ******           **** **
NaJAZj            CAAAAGAGAAAAAAATAGAATTCAAGCAACTTCTCCTTATCATCGCTAG 399
```

I. Sequence alignment of NaJAZk.1 and ir-construct of NaJAZd

```
ir-construct      ------------------------------------------------------------
NaJAZk.1          ATGCCGCCGGAAGAATCAGTTTCGAAGTCACTTCTCGACAAGCCGCTTCAGCAACTCACT 60


ir-construct      -------------------------------------------ACTACAAAATCTGAACC 17
                                                             **  **** *   *
NaJAZk.1          GAAGATGACATTTCTCAGCTCACTCGCGAAGATTGCCGCCGTTACCTCAAACTTAAAGGT 120

ir-construct      A-GAAAAGGC---------ACAAATGACA----------------ATATT---------- 41
                  * ** *****         * **** ***                ****
NaJAZk.1          ATGAGAAGGCCGTCATGGAATAAATCACAGGCGATTCAGCAAGTGATATCACTGAAGGCT 180

ir-construct      ----TTATGGTG--GTCAAGTTATT------GTATTTG-ATGA---TTT----------- 74
                      **  *  *  * **  **         * * * * * **   ***
NaJAZk.1          CTCCTTGAGACGACGCCAGATTCCGACGCCGGCACTCGGAAGAAACTTTACATTCCTCGC 240

ir-construct      TCCAGCA----GCTA--------------AGGCAAA--------TGAAA---TCATGAAA 105
                  ***  **    * **              *** ***        ****    *  **  **
NaJAZk.1          TCCGACACTAAGTTACACCATGTCCAGAGAGGGAAAAATACTGATGAAGAATTTATCGAA 300

ir-construct      TTGGCTAGCAAGAAA-----------AACAACA--ACAACAACAAACAGA---------- 142
                  ******    *** *           ** ****  ***  *  ** * **
NaJAZk.1          TTGGCTGAAGAGACAGTGCCTGATGGAAGAACACTACAGGATAAAGCTGATCTTTCCGGC 360

ir-construct      -------------ATTTGG------CTACTAACATATTTTCTTATCCTA----------- 172
                               ** ***      *** * * * **   **  * ***
```

```
NaJAZk.1          GATGCAACTCCCAATCTGGTTGCTGCTATTGATAAATCAGCTCCTTCTAGAACAATAGGT 420


ir-construct      -TGGTAAATA-ATCA-------AAATT-----------CAGC-------TGAAT------ 199
                     *** *** ****       ****            ****       *****
NaJAZk.1          TCAGTAGATACATCAGCAGGACAAATGACAATCTTCTACAGCGGCAAGGTGAATGTCTAT 480

ir-construct      ----------CTGTT--------ACTACCAAT--------TTGACTCAAGAAC--TTCGT 231
                            *** *        ** * ****        ***  *****  *  ** **
NaJAZk.1          GATGATGTGCCTGCTGACAAGGCACAAACAATCATGCGTGTTGCTTCAAGCTCCCTTTGT 540

ir-construct      TCGC------GAACTC----ACGTGCCAATATCACAA-TCATCTGT-----TGCTGATTT 275
                    **       *****       *** * * *  **  *** *  *     *****  **
NaJAZk.1          GTGCCTTCAGAAACTCCATTGAATGCTACTGTAGCAGCTCAACATTCAACATGCTG-CTT 599

ir-construct      ACCGATTGCGAG---------ACG---AAATTC---------ACTT-------------- 300
                  ** * **** *          ***   * ****         ****
NaJAZk.1          ACAGGTTGCAAATACTAAACTACGCCCAGATTCTGATATGGTACTTCTGCCGACTATTCA 659

ir-construct      -ACA-------------------------------------------------------- 303
                   ***
NaJAZk.1          AACAGAAGCAGTAGAGAACCCCTCAAGCAGGAAAGCATCAGTACAACGATATCTTGAGAA 719

ir-construct      ----------------
NaJAZk.1          GCGAAAAGACAGGTGA 735
```

J. Sequence alignment of NaJAZl and ir-construct of NaJAZd

```
ir-construct      ---------------------------------------ACTACAAAATCTGA------- 14
                                                         * *  *** * * *
NaJAZl            ATGTATTGCAGCTCCAAAGTGGCAAATAATTTCTTGAAGATTGAAAAGTTTAATAAAAAC 60

ir-construct      ----ACCAGAAAAGGCACA--AATGACAATATTTTA-----------------TGGTGGT 51
                      *  *  * * *** *  ***** *  *  ***                 **** **
NaJAZl            TTCGATTATCAGAAGCAAATTAATGAAAGCAACTTAAAAGGTATAGGTAACAATGGTAGT 120

ir-construct      CA------AGTTATTGTATT-----TGATGATTTT-CCAG-----------------CAG 82
                  **      * *   **   *     **   ***** ****                 ***
NaJAZl            CACAGAAGAATGTCTGCTCTAGAGCTGGCAATTTTACCAGGAATCAAGCATGATACTCAG 180

ir-construct      CT------------AAGGCAAATG---AAATCATGA-AATTGGCTA--------GCAAGA 118
                  **            ***  * * *    *****  * * **  ***        **  *
NaJAZl            CTTCCTGTTTTACAAAGAGAGAAGTCTGAATCAGAACAGTTAACTATATTCTATGCTGGG 240

ir-construct      A--AAACA-----ACAACAAC----------AACAAACA-----------GAATTTGGCT 150
                  *    ***     *  ****           ** *  **           * **** **
NaJAZl            ATTGTACATGTTTATGACAATCTTCCTGTTGAAAAGGCACAGTCTATTATGGATTTTGCA 300

ir-construct      ACTAACA------TATTTTCTTATCCTA----TGGTAAAT-------------------- 180
                    * * *      * ******  * ***    **  ****
NaJAZl            CGTGAAAGTTCATTGTTTTCTGGTTCTACTAATGTAAAATTTCCCCCCAAAGAAGCTGAA 360

ir-construct      ---AATCAAAATTC---AGCTGAATCTG--------TT---------ACTACCAATT--- 214
                     ***** ** **   ** *  ** **        **         *** ******
NaJAZl            CCAAATCAGAAATCCCAAGTTCCATTTGCGTGCAAGTTCCAAGCAGAACTGCCAATTGCC 420

ir-construct      ---------TGACTCAAGAACTTCGTTCGCGAACTCACGTGCCAATATCACAA------- 258
                           *  ** ****  *** **** ***    * *  **  ** * **
NaJAZl            AGAAGGAAATCCCTGAAGAGATTC-TTCGAGAAACGCCATAACAGGATTATAAGTAAACA 479
```

```
ir-construct      ----------TCATCTGTTGCTGATTTAC-------CGATTGC--------GAGACGAAA 293
                            **  *****  *  *  **        ** **         ***  ****
NaJAZl            CCCTTATGCCTCTCCTGTTATTACTCAACATGAAGATGAATGTAATGATCAGAGTGGAAA 539
ir-construct      TT-CACTT------------ACA----- 303
                  **  * **            * *
NaJAZl            TTATAGTTTGAAGGAAAAGAATAGTTAA 567
```

K. Sequence alignment of NaJAZm and ir-construct of NaJAZd

```
ir-construct      ACTACAAAATCTGAACCAGAAAAGGCACAAATGACAATATTTTATGGTGGTCAAGTTATT 60
                     *  **        *****  ***    *         * *** ** ***   ** **
NaJAZm            ---ATGAAGCA-----CAGAATTGGCCTTCAACTTTTCCCTCTATTGTCGTC---TTCTT 49

ir-construct      GTATTTGATGATTTTCCAGCAGCTAAGGCAAATGAAATCATGA---AATTGGCTAGCAAG 117
                   *  ** *   **   *** **         ** *     **      * * *** ****
NaJAZm            CTTCTTCAGAGTTCCACAGTAGT-------GATCACTCTGTGGCCTGCTCGTCTA-CAAG 101

ir-construct      AAAA------ACAA----CAACAACA-ACAAAC---AGAATTTGGC-TA-CTAACATAT- 160
                  ***       ****    * *****  **** *   ***** **   ** ** *  *
NaJAZm            AAAGCATATCACAATTTTCTACAACGCACAAGCTGGAGAATGTGATATATCTGAAGTGCA 161

ir-construct      ----TTTCTTATCCTATGGTA-----AATAATCAAAATTCAGC--TGAATCTGTTACTAC 209
                      **    *** ***** *      * **  *****  *    **   ****  **
NaJAZm            GGCCTTAGCCATCTTATGGCATGCAAGACAAGTAAAATATAATAATGGG-CTGTCCCTGA 220

ir-construct      CAATTTGACTCAAGAACTTCGTTCGCGAACTCACGTGCCAATATCACAATCATCTGTTGC 269
                   **  *  **  ***  **  *  ** **   *   *  **      * ***   *  **
NaJAZm            GAAAATCCCTACAGAGGTT--TCTGCAAAAG-AGAAGAGAAAGGAGGATTCAA--GCAGC 275

ir-construct      TGATTTACCGATTGCGAGACGAAATTCACTTACA 303
                  * **  *     * * **
NaJAZm            TAATCCA-----TACTAG---------------- 288
```
